# Multiplex Imaging Mass Cytometry Reveals Prognostic Immunosuppressive Subpopulations and Macrophage-Driven Metastasis in Osteosarcoma

**DOI:** 10.3390/cancers17172780

**Published:** 2025-08-26

**Authors:** Benjamin B. Gyau, Junyan Wang, Weiguo Wu, Brooks Scull, Angela M. Major, Weidong Jin, Justin M. M. Cates, John Hicks, Tsz-Kwong Man

**Affiliations:** 1 Section of Hematology and Oncology, Department of Pediatrics, Baylor College of Medicine, Houston, TX 77030, USA; benjamin.gyau@bcm.edu (B.B.G.); junyanw779@gmail.com (J.W.);; 2 Texas Children’s Cancer and Hematology Center, Houston, TX 77030, USA; 3 Section of High Parameter Cytometry, Cytometry and Cell Sorting Core, Baylor College of Medicine, Houston, TX 77030, USA; weiguo.wu@bcm.edu; 4 Section of Histological Research and Development, Department of Pathology, Texas Children’s Hospital, Houston, TX 77030, USA; 5 PathGroup Laboratories, Nashville, TN 37217, USA; 6 Section of Pathology and Immunology, Department of Pathology, Baylor College of Medicine, Houston, TX 77030, USA; 7 Dan L. Duncan Comprehensive Cancer Center, Baylor College of Medicine, Houston, TX 77030, USA

**Keywords:** osteosarcoma, metastasis, imaging mass cytometry, tumor microenvironment, immunosuppressive cells, spatial analysis, M2 macrophages, immune-tumor crosstalk, MIP-1α, migration

## Abstract

**Background:** Metastasis continues to be a leading cause of mortality in osteosarcoma (OS) among pediatric and young adult populations, with few effective therapeutic options available. Despite immunotherapy advancements, its efficacy in OS is hindered by an incomplete understanding of the immunosuppressive tumor microenvironment (TME). **Methods:** We utilized multiplex imaging mass cytometry and phenoplexing to characterize immune and stromal cell populations within the TME of a tissue microarray comprising 51 primary OS tumors. The prognostic significance of TME cell abundance and spatial cell–cell distance was evaluated using Kaplan–Meier and Cox regression analyses. To investigate macrophage functionality in vivo, we employed orthotopic xenograft mouse models by co-injecting THP-1-derived M0 or M2 macrophages with 143B OS cells to assess their impact on tumor growth and pulmonary metastasis. Mechanisms of macrophage-mediated metastasis were explored using Luminex, ELISA, and transwell migration assays. **Results:** Our results showed that macrophages dominated the TME, with M0 and M2 subtypes significantly outnumbering M1 macrophages (M1) and other myeloid cells. T cells and myeloid-derived suppressor cells (MDSC) were the second and third most abundant immune populations, respectively. Among stromal cells, endothelial cells predominated over fibroblasts. While individual immunosuppressive cell populations (M2, MDSC, and Treg) showed no direct correlation with clinical outcomes, the collective abundance of M2 and MDSC was significantly associated with reduced metastasis-free survival (MFS, *p* = 0.0244) and recurrence-free survival (RFS, *p* = 0.0040). Notably, closer spatial proximity between M2 macrophages and immunosuppressive cells (*p* = 0.0248) or Ki-67^+^ cells (*p* = 0.0321) correlated with decreased MFS, suggesting the formation of an M2-centric immunosuppressive and pro-tumor hub. In vivo, co-injection of M2 macrophages with 143B cells significantly enhanced pulmonary metastasis (*p* = 0.0140). Luminex analysis identified M2-derived MIP-1α (CCL3) as a candidate chemokine driving OS cell metastatic potential. **Conclusions:** This study provides a high-resolution map of the OS TME, highlighting the prognostic significance of M2 and immunosuppressive cell interactions in driving metastasis, potentially through MIP-1α signaling. These findings establish a foundation for developing targeted immunotherapies to improve outcomes in metastatic OS.

## Simple Summary

Osteosarcoma is a type of bone cancer that mostly affects kids and teens. The biggest danger is when the cancer spreads to the lungs, and current treatments do not work well for those cases. Immunotherapy, which helps the body’s immune system fight cancer, has worked for other cancers but is limited for osteosarcoma because we do not fully understand the environment around the tumor. Our research looked at various cells around the tumor to study why patients have worse outcomes. We found that certain cells, called M2 macrophages (M2) and myeloid-derived suppressor cells (MDSC), which weaken the immune system, are very common in these tumors. When there are a lot of these cells, the cancer is more likely to come back. Even worse, when these immune-suppressing cells are very close to M2 in the tumor, the cancer is much more likely to spread to the patient’s lungs. We further showed that M2 helps cancer spread to the lungs in mice. We also discovered that M2 releases a protein called MIP-1α (CCL3), which enhances the ability of cancer cells to spread. These findings could help create new treatments to target these cells and stop the cancer from spreading.

## 1. Introduction

Osteosarcoma (OS), the most common primary bone malignancy in children, adolescents, and young adults, affects approximately 4–5 per million individuals annually, comprising 2–3% of pediatric cancers [1]. Since the 1980s, advancements in surgical techniques and multi-agent chemotherapy regimens, including doxorubicin, cisplatin, and methotrexate, have elevated 5-year survival rates for localized OS to 60–70% [2,3]. However, patients with relapsed or metastatic (R/M) disease, particularly those with pulmonary metastases, face significantly worse prognoses, with 5-year survival rates ranging from 10 to 25% [4,5,6]. The lack of effective therapies for R/M OS results in uniform treatment protocols applied across all patients, regardless of clinical risk stratification. Genomic studies reveal frequent mutations in TP53 and RB, which are challenging to target due to their roles in tumor suppression and chromatin remodeling [7]. These molecular barriers, coupled with chemoresistance and metastatic progression, have led to stagnant survival improvements over the past four decades, underscoring the urgent need for novel therapeutic strategies tailored to R/M OS.

Cancer immunotherapy, particularly immune checkpoint inhibitors targeting the programmed cell death protein 1 (PD-1)/PD-L1 axis, has revolutionized treatment for cancers such as melanoma, non-small cell lung cancer, and renal cell carcinoma. Phase III trials report improvements in overall survival and durable responses in patients [8,9]. In contrast, immunotherapy has shown limited efficacy in OS. Pembrolizumab and nivolumab, PD-1 inhibitors, achieved low objective response rates in phase II trials for relapsed or metastatic OS [5,10]. This resistance is largely attributed to the immunosuppressive tumor microenvironment (TME) of OS, a dynamic ecosystem comprising immune cells, stromal cells, extracellular matrix components, and soluble factors that collectively promote immune evasion and tumor progression [11]. Immunosuppressive cells (IMSC), including M2 macrophages (M2), myeloid-derived suppressor cells (MDSC), and regulatory T cells (Treg), dominate the OS TME, suppressing effector T cell function and blunting immunotherapy efficacy [12,13].

The success of immunotherapy is critically dependent on the TME’s cellular composition, spatial organization, and intercellular interactions. Ligon et al., utilizing functional cytometry and immunohistochemistry, identified an immunosuppressive niche at the periphery of OS pulmonary metastases, enriched with M2 macrophages (M2); polymorphonuclear MDSC; and LAG-3-, TIM-3-, and PD-L1-expressing tumor-infiltrating lymphocytes (TILs), which correlated with worse progression-free survival [13]. Similarly, Lacinski et al. demonstrated that increased MDSC–PD-1^+^ T cell interactions and immune-cold parenchyma in OS were associated with a reduction in 5-year survival [14]. These findings highlight the pivotal role of spatially organized immunosuppressive networks in driving clinical outcomes in OS. We reason that a comprehensive characterization of the immune and stromal cell populations in the TME, including their abundance and spatial relationships, will likely reveal novel prognostic biomarkers and therapeutic targets to improve the treatment for R/M OS.

In this study, we employed multiplex imaging mass cytometry (IMC) to profile the cellular composition and spatial distribution of diverse immune and stromal cell populations in 51 primary OS tumors. We observed heterogeneous distributions of immune and stromal cells, and the abundance of IMSC and their spatial distance from M2 significantly correlated with worse outcomes. We further demonstrated that M2 increased pulmonary metastases in an orthotopic xenograft mouse model and identified M2-derived MIP-1α (CCL3) as a key cytokine driving the metastatic propensity of OS cells.

## 2. Materials and Methods

### 2.1. TMA and Patient Characteristics

A tissue microarray (TMA) comprising 58 formalin-fixed paraffin-embedded (FFPE) pretreatment primary OS biopsy samples was provided by Dr. Justin Cates (then at Vanderbilt University Medical Center). One tumor core per case was used in IMC. The diameter of each core was 1.0 mm (total analyzable area = 0.785 mm^2^). The samples were obtained from diagnostic or incisional biopsies collected prior to chemotherapy or surgical intervention. Most patients (56%) received combination therapy with cisplatin, adriamycin, and/or methotrexate, with some variations that included additional agents (e.g., ifosfamide) or unspecified regimens. Diagnoses were rendered by pathologists in College of American Pathologists-certified laboratories, according to WHO and Children’s Oncology Group criteria. Clinical and demographic data, including sex, race, age at diagnosis, metastatic status, disease recurrence events, and treatment modalities (e.g., chemotherapy regimens and surgical resection), are summarized in Table 1, and the detailed data are provided in Supplementary Table S1.

### 2.2. Tissue Antibody Staining and IMC Acquisition

Before undergoing the IMC analysis, the TMA was verified to contain viable tumor tissue, and its histomorphology was validated using H&E staining. Representative images are shown in Supplementary Figure S1. The TMA was then stained with a panel of 33 metal isotope-conjugated antibodies and an intercalator-191Ir/193Ir nuclear stain (Supplementary Table S2), designed and validated by the Cytometry and Cell Sorting Core (CCSC) at Baylor College of Medicine, to characterize diverse cell populations (e.g., myeloid, lymphoid, and stromal cells), cellular states (e.g., proliferation and apoptosis), and extracellular matrix components (e.g., COL1). Stained TMAs were imaged using the Hyperion Imaging Mass Cytometry (IMC) system (Standard BioTools, South San Francisco, CA, USA). A schematic overview of the imaging mass cytometry (IMC) workflow utilized in this study is provided in Supplementary Figure S2. Briefly, TMA slides were baked at 60 °C for 2 h, deparaffinized in three 5 min washes with 100% xylene, and rehydrated through a graded ethanol series (100%, 100%, 95%, and 70%) for 5 min each, followed by a 5 min wash in deionized water with gentle agitation. Antigen retrieval was performed by incubating slides in 300 mL Diva Decloaker buffer (Biocare Medical, Pachico, CA, USA) in a pressure cooker at 110 °C for 15 min, cooling to 70 °C, and washing in deionized water for 10 min and PBS for 10 min with gentle agitation. Slides were blocked with 3% bovine serum albumin (BSA) in PBS for 45 min at room temperature (RT) in a humidified chamber, then incubated overnight at 4 °C with the antibody cocktail diluted in PBS with 0.5% BSA. Slides were washed twice for 8 min each in PBS-T (PBS with 0.1% Tween-20) and PBS with slow agitation at RT, followed by staining with 125 µM Intercalator-Ir (Standard BioTools) diluted 1:1000 in PBS for 30 min at RT in a humidified chamber. After a 5 min wash in deionized water with gentle agitation, slides were air-dried for at least 20 min at RT. For IMC acquisition, slides were loaded into the Hyperion tissue imager (Standard BioTools), where regions of interest (1 mm^2^/pixel) were ablated using a high-energy UV laser at 200 Hz. Ablated material was transported via argon gas into the mass cytometer’s plasma, where it was atomized, ionized, filtered, and analyzed using time-of-flight (TOF) mass spectrometry to generate high-resolution spatial data.

### 2.3. Image Processing, Cell Segmentation, and Quality Control

Following IMC acquisition, multiplexed image data with spatial information were reconstructed and stored in .mcd file format for downstream analysis. Initial cell-level quality control (QC) was performed using the MCD Viewer software (V1.0.560.6; Standard BioTools) to assess image integrity. Full-resolution IMC images (10× magnification) in .mcd format were imported into the Visiopharm^®^ platform (Visiopharm A/S, Hørsholm, Denmark) and color-adjusted to optimize visualization for nuclear detection and single-cell segmentation (Supplementary Figure S2). A detailed protocol for app creation, configuration, cell segmentation, algorithm training, and phenotyping in Visiopharm has been reported [15]. Single-cell segmentation utilized DNA signals (Intercalator-Ir) to identify nuclei, followed by Visiopharm’s polynomial blob detection algorithm to refine cell boundary delineation and ensure accurate separation of adjacent cells. Cases and markers underwent rigorous QC to ensure data reliability. Tumor cores were evaluated for nuclear and marker staining quality. Seven tumor cores exhibiting poor nuclear staining (low or diffuse Ir191 intensity) were excluded from downstream analyses (Supplementary Figure S3). The percentage of Ir191^+^ cells (indicating successful nuclear staining) relative to total nuclear-segmented cells was calculated per core. Cores with <25% Ir191^+^ cells, representing an inflection point for poor staining quality, were excluded (Supplementary Figure S3). For marker-level QC, eight antibodies (T-bet, CD38, CD19, CTLA-4, CD86, PD-L1, CD33, and Vimentin) showing suboptimal staining were removed (Supplementary Figure S3). A case was considered positive for a marker if ≥3 cells per core stained positive. Markers were excluded if <10% of cases were positive, ensuring only robustly expressed markers were analyzed. These QC steps ensured high-quality data for subsequent cellular and spatial analyses of the OS TME.

### 2.4. Cell Phenotyping and Spatial Analysis

After quality control, cell phenotyping was conducted on 51 OS tumor cores using the Visiopharm^®^ Phenoplex Guided Workflow (Phenoplex tool, version 2024.07, Visiopharm A/S). A detailed step-by-step protocol for phenotyping and configuration is available [15]. Phenotyped data, including all cells and their assigned phenotypes, were exported as .tsv files for downstream analyses, encompassing cell positivity data and spatial metrics. Immune and stromal cell population abundances were quantified as the percentage of each cell type relative to the total number of detectable cells in each tumor core. The immunosuppressive cell (IMSC) abundance per core was calculated as the mean of the percentages of M2, myeloid-derived suppressor cell (MDSC), and regulatory T cell (Treg) counts per case. The marker combinations used for phenotyping TME cell populations are listed in Supplementary Table S3. Spatial analysis was performed using the Phenoplex Guided Workflow to evaluate the spatial distribution of TME cells within a 250 µm radius of M2. Specific markers for M2 (e.g., CD68^+^CD163^+^), MDSC (e.g., CD11b^+^CD14^+^CD15^−^ and CD11b^+^CD14^−^CD15^+^), and Treg (e.g., CD3^+^FoxP3^+^) were defined to annotate these populations. For each core, median distances were calculated for M2–M2, M2–MDSC, and M2–Treg pairs within the specified radius. The M2–IMSC distance per core was determined as the mean of these median distances, reflecting the average spatial proximity of immunosuppressive cells to M2. Cell density relative to M2 macrophages per tumor core was calculated as the median cell count of each cell type within a 250 µm radius of M2, divided by the area of the region. For collective cell populations, such as IMSC, the total cell count around M2 per tumor core was the sum of the median counts of the constituent cell types. These metrics enabled assessment of immunosuppressive cell communities and their prognostic implications in the OS TME.

### 2.5. Mice and OS Cell Lines

Eight-to-sixteen-week-old NOD.CB17-Prkdc scid/J (NOD/SCID) mice were obtained from The Jackson Laboratory (Bar Harbor, ME, USA) and housed in a pathogen-free facility at Baylor College of Medicine, Houston, TX, USA. The weight of mice prior to experiments was 18–25 mg. Human OS cell lines 143B, LM7, and MG63.3, and the THP-1 monocytic cell line, were acquired from the American Type Culture Collection (ATCC, Manassas, VA, USA) and stored in liquid nitrogen until use. OS cell lines were cultured in Dulbecco’s Modified Eagle Medium (DMEM; Gibco brand, Thermo Fisher Scientific, Waltham, MA, USA) supplemented with 10% fetal bovine serum (FBS; Gibco, Thermo Fisher Scientific) and 1% penicillin/streptomycin (100 U/mL of penicillin, 100 µg/mL of streptomycin; Gibco, Thermo Fisher Scientific). THP-1 cells were maintained in RPMI 1640 medium (Gibco, Thermo Fisher Scientific) supplemented with 10% FBS, 10 mM HEPES, 1 mM sodium pyruvate, 2.5 g/L of D-glucose, 50 µM β-mercaptoethanol, and 1% penicillin/streptomycin (all Gibco brand, Thermo Fisher Scientific), hereafter referred to as macrophage maintenance medium (MMM). All cell lines were incubated at 37 °C in a humidified 5% CO_2_ atmosphere.

### 2.6. Macrophage Polarization

To investigate the effects of macrophages on OS cell growth and metastasis, we utilized the THP-1 monocytic cell line (ATCC, Manassas, VA, USA) as a model for macrophage differentiation. THP-1 cells, when treated with phorbol-12-myristate-13-acetate (PMA; Sigma-Aldrich, St. Louis, MO, USA), activate protein kinase C (PKC), differentiating into macrophage-like cells with increased adherence, reduced proliferation, and phenotypic resemblance to mature human macrophages [16]. THP-1 cells were cultured in MMM at 37 °C in a 5% CO_2_ humidified atmosphere until the log phase (approximately 7 days post-thaw from liquid nitrogen storage). Cells were seeded at 0.8–1 × 10^6^ cells per well in 6-well plates or 0.8–1 × 10^7^ cells in 75 cm^2^ flasks and differentiated with 10 ng/mL PMA for 48–72 h, yielding M0 macrophages (M0) characterized by adherence and loss of proliferative capacity. M0 macrophages were cultured in fresh MMM for 5 days to enhance cytoplasmic volume and functional similarity to naïve human macrophages, followed by a 24 h rest in fresh MMM to stabilize their phenotype. For polarization, M0 cells were treated for 48 h with either (1) 20 ng/mL of interferon-gamma (IFN-γ; MilliporeSigma, Burlington, MA, USA) and 10 pg/mL of lipopolysaccharide (LPS; MilliporeSigma) to induce M1 macrophages, or (2) 20 ng/mL of interleukin-4 (IL-4; R&D Systems Inc., Minneapolis, MN, USA) and 20 ng/mL of interleukin-13 (IL-13; R&D Systems Inc.) to induce M2. To ensure high polarization efficiency, particularly for M2, fresh cytokine cocktails were replenished for an additional 48 h. Polarized M1 and M2 macrophages were rested in fresh MMM for 24 h before use in downstream experiments, such as co-culture with OS cell lines or in vivo metastasis assays. Polarization was verified by flow cytometry or immunofluorescence for M1 (HLA-DR, BD Biosciences, Milpitas, CA, USA) and M2 (CD163, BD Biosciences) markers, as well as by ELISA for M1 (CXCL10, R&D Systems Inc.) and M2 (CCL22, R&D Systems Inc.) cytokines according to the manufacturer’s instructions.

### 2.7. Mouse Studies

To investigate the role of macrophages in OS metastasis, 2.5 × 10^5^ 143B macrophages were co-injected with 7.5 × 10^5^ THP-1-derived M0 or M2 macrophages (M0 co-culture [M0 CC] or M2 co-culture [M2 CC]) orthotopically into the proximal tibia of 8- to 16-week-old NOD. CB17-Prkdc SCID/J mice. As a control, 2.5 × 10^5^ 143B cells alone were injected without macrophages. A total of 7 mice (4 males, 3 females) were randomly assigned to each group (143B alone, M0 CC, M2 CC), totaling 21 mice, ensuring relatively equal proportions of male and female sexes. Exclusion criteria were death before or after surgery and no tumor development measured by IVIS after two weeks of intratibial injection of cells. One mouse from each co-culture group (M0 CC and M2 CC) died due to injection complications, resulting in 6 mice per co-culture group and 7 in the control group for analysis. To control for bias, indistinguishable mouse IDs were used and only known by our lab technician. Tumor volume and metastatic burden were quantified by an independent assessor. Tumor growth was monitored weekly with calipers starting approximately two weeks post-injection, when tumors became palpable. Five weeks post-injection, mice were euthanized via CO_2_ asphyxiation followed by cervical dislocation, per IACUC guidelines. Lungs were harvested, fixed in 10% neutral buffered formalin, and processed for histopathological examination. Hematoxylin and eosin (H&E)-stained lung sections were analyzed to quantify total pulmonary metastases under a light microscope.

### 2.8. OS–Macrophage Co-Culture and Cytokine Analysis

To identify differentially expressed soluble factors in OS–macrophage interactions, non-contact co-cultures of THP-1-derived M0, M1, and M2 macrophages with OS cell lines (143B, MG63.3, LM7) were established using 6-well transwell plates (0.4 µm pore size; Corning Inc., Kennebunk, ME, USA). Macrophages were seeded at 3 × 10^5^ cells/well in the upper insert in MMM, while OS cells were seeded at 1 × 10^5^ cells/well in the lower well in DMEM with 10% FBS and 1% penicillin/streptomycin, maintaining a 3:1 macrophage-to-OS cell ratio. Mono-cultures of macrophages and OS cells served as controls. After 72 h of co-culture at 37 °C in a 5% CO_2_ humidified atmosphere, conditioned media (CM) were collected, centrifuged at 21,000× *g* for 5–10 min at 4 °C to remove debris, and stored at −80 °C. Co-cultured cells were trypsinized, washed twice in PBS, pelleted by centrifugation at 250× *g* for 5 min, and stored at −80°C until downstream analyses.

Cytokine and chemokine levels in CM from mono- and co-cultures were quantified using the Milliplex MAP Human Cytokine/Chemokine Magnetic Bead Panel (HCYTA-60K-PX48, MilliporeSigma, Burlington, MA, USA), following the manufacturer’s protocol. The 48-plex assay measured levels of sCD40L, EGF, Eotaxin, FGF-2, Flt-3L, Fractalkine, G-CSF, GM-CSF, GROα, IFNα2, IFNγ, IL-1α, IL-1β, IL-1ra, IL-2, IL-3, IL-4, IL-5, IL-6, IL-7, IL-8, IL-9, IL-10, IL-12p40, IL-12p70, IL-13, IL-15, IL-17A, IL-17E, IL-17F, IL-18, IL-22, IL-27, CXCL10, MCP-1, MCP-3, M-CSF, MDC, MIG, MIP-1α, MIP-1b, PDGF-AA, PDGF-AB/BB, TGFα, TNFα, TNFβ, and VEGF-A. Briefly, 25 µL of CM samples, standards, or controls were incubated with 25 µL of antibody-coated magnetic beads, assay buffer, and matrix solution overnight at 4 °C with gentle orbital shaking (300 rpm). Following bead capture, plates were washed 3 times using a magnetic plate washer, and detection antibodies were added for 1 h at RT. Streptavidin–phycoerythrin was then applied for 30 min at RT, followed by 3 additional washes. Plates were analyzed on a Bio-Plex 200 system (Bio-Rad, Hercules, CA, USA) using Bio-Plex Manager software (version 4.1). At least 50 beads per analyte were collected, and median fluorescence intensities (MFI) of the beads were measured. The MFIs were then background-subtracted before statistical analysis. All measurements were duplicated.

### 2.9. Luminex Data Validation and Protein Source Verification

To validate the differential abundance of MIP-1α (CCL3) identified in the Luminex assay, two independent experiments were conducted, quantifying MIP-1α levels in CM from OS-M2 co-cultures and mono-cultures using an enzyme-linked immunosorbent assay (Human CCL3/MIP-1α ELISA Kit, R&D Systems Inc.) per the manufacturer’s instructions. ELISA was also performed on cell lysates from corresponding cell pellets to confirm the cellular source of MIP-1α in co-cultures (e.g., M2 vs. OS cells). For lysate preparation, cultured cells were detached with 0.25% trypsin-EDTA (Gibco brand, Thermo Fisher Scientific) for 5–10 min at 37 °C, washed twice with PBS, and pelleted via centrifugation at 250× *g* for 5 min at 4 °C. Pellets were suspended in 1X RIPA buffer (Santa Cruz, Dallas, TX, USA) supplemented with protease and phosphatase inhibitor cocktails (Cell Signaling, Danvers, MA, USA), incubated on ice for 30 min with intermittent vortexing, and centrifuged at 21,000× *g* for 15 min at 4 °C. The supernatant (cell lysate) was collected and stored at −80 °C until analysis. ELISA was performed on both CM and lysates, with absorbance measured at 450 nm using a MiniMax 300 Imaging Cytometer (Molecular Devices, San Jose, CA, USA). MIP-1α concentrations were calculated based on a standard curve, with samples run in duplicates.

### 2.10. Cell Migration Assay

The cell migratory effects of recombinant human MIP-1α (CCL3) protein (R&D Systems Inc.) and CM from co-cultures of THP-1-derived M2 with OS cell lines (143B, MG63.3, LM7) were assessed using the QCM 24-Well Cell Migration Assay Kit (ECM508, MilliporeSigma, Burlington, MA, USA), following the manufacturer’s protocol. OS cells were serum-starved overnight in DMEM with 0.1% FBS to minimize proliferation and baseline migration. For each assay, 5 × 10^4^ cells in 300 µL of FBS-depleted DMEM (0.1% FBS) were seeded into the upper chamber of a 24-well transwell insert. The lower chamber contained 500 µL of FBS-depleted DMEM as a baseline control. For the recombinant MIP-1α assay, cells in the upper chamber were treated with 0.1 µg/mL or 1 µg/mL of MIP-1α diluted in FBS-depleted DMEM. For the CM assay, co-culture CM was centrifuged at 21,000× *g* for 5–10 min at 4 °C to remove debris, and 5 × 10^4^ OS cells were suspended in 300 µL of CM before seeding in the upper chamber. After 4 h of incubation at 37 °C in a 5% CO_2_ humidified atmosphere, migrated cells on the lower surface of the insert were fixed and stained with crystal violet (Cell Stain Solution, MilliporeSigma, Burlington, MA, USA). Inserts were imaged at 10× magnification using an EVOS FL Microscope (Invitrogen, Waltham, MA, USA). Migrated cells were quantified using ImageJ software (version 1.53e; Java 1.8.0_172, NIH, Bethesda, MD, USA) by counting cells in five random fields per well, with results averaged per replicate. Experiments were performed at least in triplicate, with three independent biological replicates to ensure reproducibility. Data were expressed as the mean number of migrated cells per field ± standard deviation.

### 2.11. Statistical Analyses

Differences in cell type abundance between two cell populations or subpopulations were evaluated using a paired *t*-test, while comparisons across three or more cell populations or subpopulations were analyzed with a repeated measures one-way analysis of variance (ANOVA) followed by Tukey’s post hoc test for pairwise comparisons. For survival analyses, cases with initial metastasis were excluded, resulting in 43 localized cases with clinical information for the analysis (Supplementary Table S1). Metastasis-free survival (MFS) and recurrence-free survival (RFS) were assessed using the Kaplan–Meier method, with cell type abundance (%), and spatial distances or cell densities to M2 (e.g., M2–IMSC proximity) stratified into binary categorical variables. Median values served as cutoffs to classify abundance or densities as “high” or “low” and distances as “near” or “far”. MFS was defined as the time from diagnosis to the first documented distant metastasis or the last follow-up if no metastasis occurred. RFS was defined as the time from diagnosis to the first local or metastatic recurrence or last follow-up if no recurrence occurred. Significance of survival differences was computed using a log-rank test, while individual Hazard Ratios (HRs) were computed with a COX proportional hazards model.

Differences in pulmonary metastatic burden and tumor growth in the orthotopic xenograft model were analyzed using a two-tailed Mann–Whitney U test. Cytokine and chemokine concentrations in CM from OS–macrophage mono- and co-cultures were compared using two-way ANOVA (culture conditions and cell lines) with Tukey’s post hoc test. Differentially abundant cytokines were defined as significantly different between co-cultures and mono-cultures in at least two of three cell lines. For the cell migration assay, differences in migrated cell counts across treatment conditions (e.g., MIP-1α concentrations, CM) were assessed with an ordinary one-way ANOVA followed by Tukey’s post hoc. All statistical analyses were performed using GraphPad Prism (version 10.1.0 (316); GraphPad Software, San Diego, CA, USA). A *p*-value < 0.05 was considered statistically significant, with exact *p*-values reported in the text unless otherwise stated.

## 3. Results

### 3.1. Cell Phenotyping Analysis of the Osteosarcoma Tumor Microenvironment

**Figure 1:**
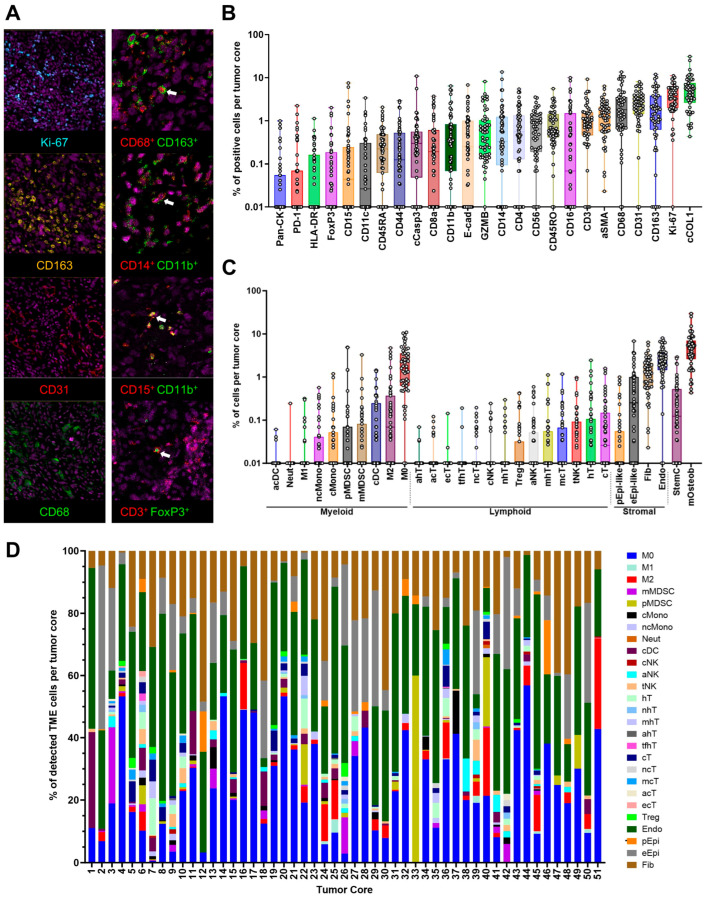
Imaging mass cytometry (IMC) and cell phenotyping analysis of OS tissue microarray (TMA). (**A**) Representative IMC images displaying nuclei, single-marker (**left**), and double-marker (**right**) staining of cells within the OS tumor microenvironment (TME). The left panel highlights the most abundant cell populations, and the right panel shows immunosuppressive cells (IMSC) with respective markers used in this study. Representative examples of double-marker-stained cells are shown with a white arrow. (**B**) Box plot illustrating the number of objects (cells) with positive staining for 25 single markers (*x*-axis) that passed quality control (QC) across 51 TMA cores (*y*-axis). The *y*-axis is presented on a log_10_ scale. (**C**) Box plot showing the percentage of various detected TME cell types following cell phenotyping across 51 tumor cores. The *y*-axis is presented on a log10 scale. (**D**) Stacked bar plot depicting the percentage of total detected immune and stromal cells per tumor core. Immune cells include CD68+ (macrophages), CD11b^+^CD14^+^CD15^−^ (mMDSC), CD11b^+^CD14^−^CD15^+^ (pMDSC), CD14^+^CD16^−^CD68^−^ (cMono), CD14^+^CD16^+^CD68^−^ (ncMono), CD15^+^CD16^+^ (neutrophils), CD11c^+^CD3^−^CD14^−^CD56^−^ (dendritic cells, DC), CD56^+^CD16^+^ (cNK), CD56^+^GZMB^+^ (aNK), CD56^+^CD3^+^ (tNK), CD3^+^CD4^+^ (helper T cells), CD3^+^CD8^+^ (cytotoxic T cells), and CD3^+^FoxP3^+^ (Treg). Stromal cells include CD31^+^ (endothelial cells), Pan CK^+^ (pan-epithelial-like cells), E-cadherin^+^ (epithelial-like cells), and aSMA^+^ (fibroblasts).

To elucidate the spatial distribution and prognostic significance of tumor-infiltrating immune and stromal cells in OS TME, we utilized imaging mass cytometry (IMC) to profile a tissue microarray (TMA) comprising 51 primary tumor samples collected at diagnosis (Table 1). Each tumor core was laser-ablated and stained with a panel of 33 metal-conjugated antibodies targeting surface and intracellular markers, designed to identify diverse cell types within the TME, including immune (e.g., macrophages and T cells), stromal (e.g., endothelial cells and fibroblasts), and other proteins, such as collagen type I (COL1) and Ki-67 (Supplementary Table S2). Representative IMC images displaying single- and double-marker staining alongside nuclear staining (Ir191) are presented in Figure 1A, with additional images in Supplementary Figure S4. A total of 111,980 Ir191^+^ nucleated cells across the 51 cases underwent cell phenotyping analysis, enabling the identification and quantification of distinct cell populations and subpopulations within the OS TME.

**Figure 2:**
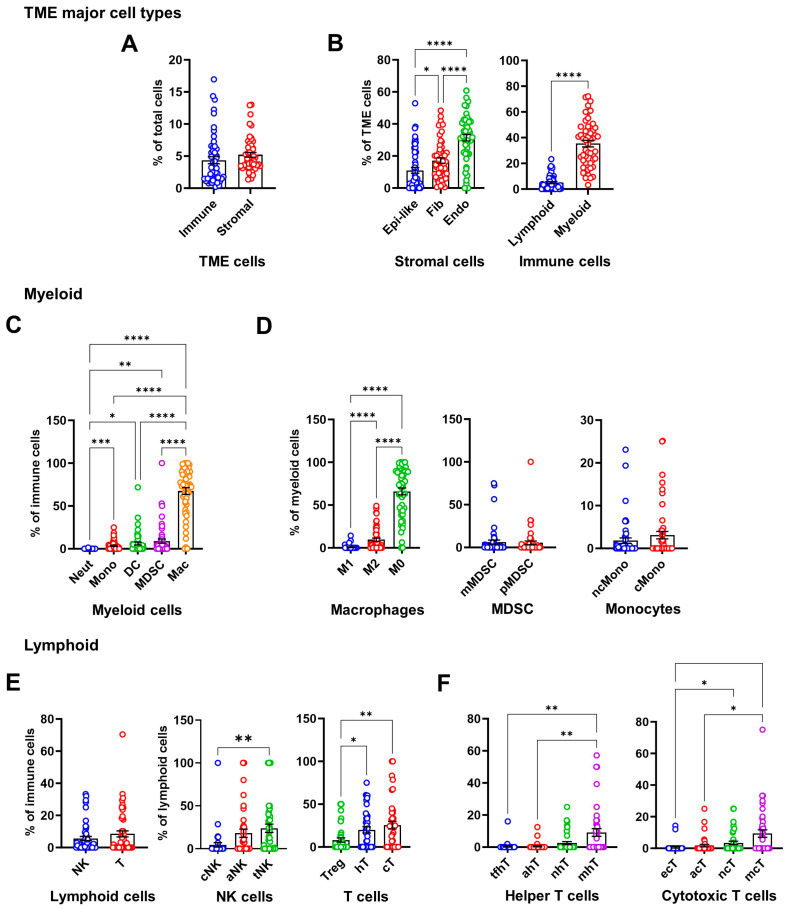
Comparisons of immune and stromal cell populations in OS tissue microarray (TMA). (**A**) Bar–scatter plot showing the percentage of total immune and stromal cells relative to total nucleated (Ir191^+^) cells in OS tumors. (**B**) Bar–scatter plot depicting the percentage of stromal (left) and immune (right) subpopulations within total tumor microenvironment cells, comprising both stromal and immune cells. (**C**) Bar–scatter plot displaying the percentage of myeloid subpopulations within total immune cells, which include both lymphoid and myeloid cells. (**D**) Bar–scatter plot illustrating the percentage of macrophage, myeloid-derived suppressor cell (MDSC), and monocyte subpopulations within total myeloid cells (sum of macrophages, mMDSC, pMDSC, cMono, ncMono, neutrophils, and dendritic cells). (**E**) Bar–scatter plot showing the percentage of lymphoid subpopulations within total immune cells (sum of cNK, aNK, tNK, hT, cT, and Treg; left). Middle: Percentage of natural killer (NK) cells (sum of cNK, aNK, and tNK) within total lymphoid cells. Right: Percentage of T cells (sum of hT, cT, and Treg) within total lymphoid cells. (**F**) Bar–scatter plot presenting the percentage of helper T (hT) and cytotoxic T (cT) subpopulations within total lymphoid cells. Each dot represents a tumor core in the OS TMA. Data are presented as mean ± SEM in bar plots overlaid by scatter plots. Statistical significance: * *p* < 0.05; ** *p* < 0.01; *** *p* < 0.001; **** *p* < 0.0001; unmarked = not significant.

Cell phenotyping analysis identified single-marker-positive cells (Figure 1B). Cells expressing collagen type 1 (cCOL1), CD68 (macrophages), CD31 (endothelial cells), CD163 (M2), or Ki-67 (proliferating cells) were most abundant (Figure 1B). COL1 is a key extracellular matrix protein [17]. Our results showed that it was predominantly expressed in cellular relative to non-cellular regions (eCOL1) (Supplementary Figure S5). Using single or multiple markers (Supplementary Table S3), we annotated the cell populations and subpopulations, with M0 and M2 macrophages, fibroblasts, endothelial cells, and stem cells ranking as the top five most abundant cell populations (Figure 1C). The proportions of various annotated cell populations in each of the OS cases are illustrated in Figure 1D. No significant difference was observed between the total proportions of immune and stromal cells in the TME (Figure 2A). Among stromal cells, CD31^+^ endothelial cells were significantly more abundant than fibroblasts and epithelial-like cells (Figure 2B, left), consistent with the role of CD31 in vascular differentiation and angiogenesis, which supports the highly metastatic nature of OS [18]. Epithelial-like cells are cells expressing epithelial markers, such as Pan-CK or E-cadherin. These findings highlight the complex cellular composition of the OS TME, with prominent immunosuppressive and vascular components driving disease progression.

OS is often characterized as an immune-cold tumor with limited infiltration of anti-tumor immune cells such as CD8^+^ cytotoxic T cells and CD56^+^ NK cells, driven by potent immunosuppressive signaling from CD68^+^CD163^+^ M2 and myeloid-derived suppressor cells (MDSC) within the TME and at the tumor periphery, respectively [13,14]. Our IMC analysis revealed a myeloid-dominated TME, with myeloid lineage cells (40% of total TME cells) significantly greater in abundance than lymphoid cells (5%) (Figure 2B, right). Within the immune cell populations, macrophages were the most abundant population (over 50% of immune cells), significantly surpassing other myeloid cells (Figure 2C). Proportions of CD68^+^HLA-DR^−^CD163^−^ M0 and CD68^+^CD163^+^ M2 were significantly higher than CD68^+^HLA-DR^+^ M1 macrophages (Figure 2D, left), while intratumoral MDSC and monocyte subtypes showed no significant differences (Figure 2D, middle and right). After macrophages, T cells and MDSC ranked as the second and third most abundant immune populations (Figure 2C, Figure 2E left; Supplementary Figure S6). Among lymphoid cells, total NK and T cell proportions were comparable (Figure 2E, left). CD56^+^ NK cell subpopulations showed higher T cell-like (CD3^+^) cell abundance compared to classical (CD16^+^) but not activated (granzyme B^+^) subtypes (Figure 2E, middle). CD4^+^ helper and CD8^+^ cytotoxic T cell proportions were similar and significantly exceeded FoxP3^+^ Treg (Figure 2E, right). CD3^+^CD4^+^CD45RO^+^ memory T helper cells (mhT) and CD3^+^CD8^+^CD45RO^+^ memory cytotoxic T cells (mcT) were significantly more abundant than other helper (tfhT, ahT) and cytotoxic (ecT, acT) subtypes, respectively, but not naïve subtypes (nhT, ncT) (Figure 2F). This high-resolution cellular map underscores the immunosuppressive, pro-tumor OS TME, with a high abundance of M2 and MDSC and sparse effector T cells.

### 3.2. Prognostic Impact of Immunosuppressive Cell Enrichment in Osteosarcoma

**Figure 3:**
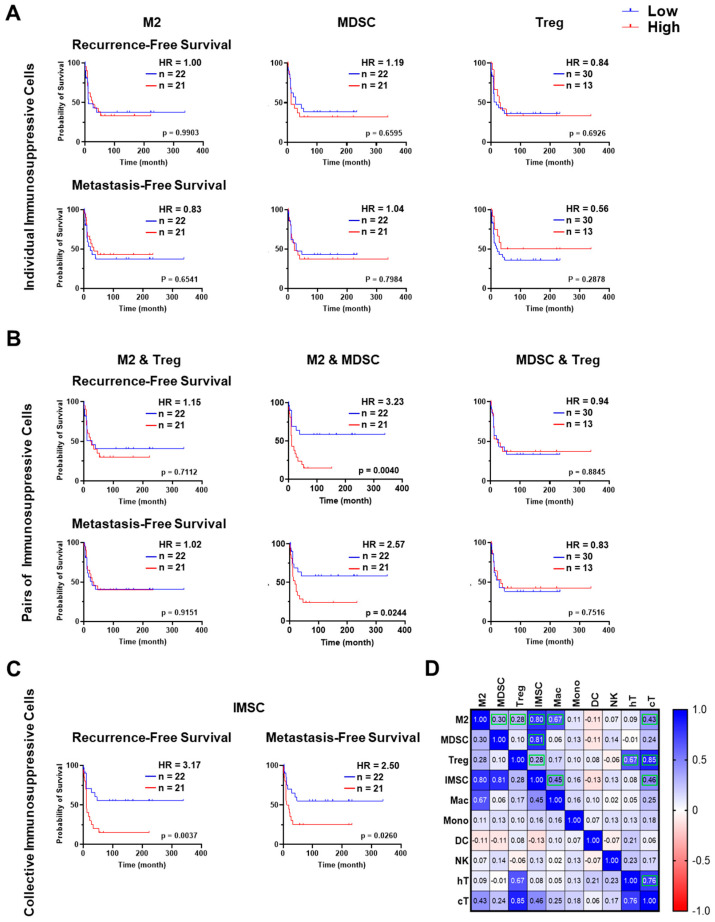
Kaplan–Meier and correlation analyses of immunosuppressive cell (IMSC) populations. (**A**–**C**) Kaplan–Meier analyses of recurrence-free survival and metastasis-free survival based on the abundances of (**A**) M2 macrophages (M2); myeloid-derived suppressor cells (MDSC); regulatory T cells (Treg); (**B**) M2 and MDSC, M2 and Treg, and MDSC and Treg; and (**C**) M2, MDSC, and Treg (IMSC) across tumor cores. Cell type abundance was quantified as the percentage of each cell type relative to the total number of detectable cells in each tumor core. These percentages were then dichotomized into a binary categorical variable, with the median percentage across all tumor cores serving as the cutoff to classify cases as “high” or “low” abundance. IMSC was calculated as the sum of the percentages of M2, MDSC & Treg. A *p*-value < 0.05 was considered statistically significant. (**D**) Pearson correlation analysis evaluating the relationship between the abundance of IMSC populations and other immune cell populations in OS TME. Green boxes indicate correlations with a *p*-value < 0.05.

To assess the prognostic significance of TME populations in OS, where metastasis and recurrence are primary causes of mortality, we analyzed their association with metastasis-free survival (MFS) and recurrence-free survival (RFS). Individual cell population alone showed no significant correlation with MFS or RFS (Figure 3A and Supplementary Table S4A), except for CD44^+^ stem cells (StemC), which correlated with worse RFS (*p* = 0.0218, HR = 2.51, Supplementary Figure S7).

However, high collective abundance of M2 and MDSC, but not M2 and Treg, or MDSC and Treg, was significantly associated with worse MFS (*p* = 0.0244, HR = 2.57, Figure 3B) and worse RFS (*p* = 0.0040, HR = 3.23, Figure 3B). Combining all three IMSC populations (M2, MDSC, and Treg) showed a similar level of significance in the outcome prediction (Figure 3C). While an inverse association between IMSC and anti-tumor immune cells such as DC, NK, and T cells is generally expected, a correlation analysis of the cell abundance in OS cases showed marked positive correlations between M2/IMSC with cytotoxic T cells, Treg, and helper or cytotoxic T cells, but an inverse correlation with DC (Figure 3D). Together, our results suggest that IMSC may negatively affect anti-tumor activities via negatively modulating the infiltration or abundance of DC, but not NK/T cells.

### 3.3. Spatial Proximity of IMSC with M2 Predicts Poorer Outcomes

**Figure 4:**
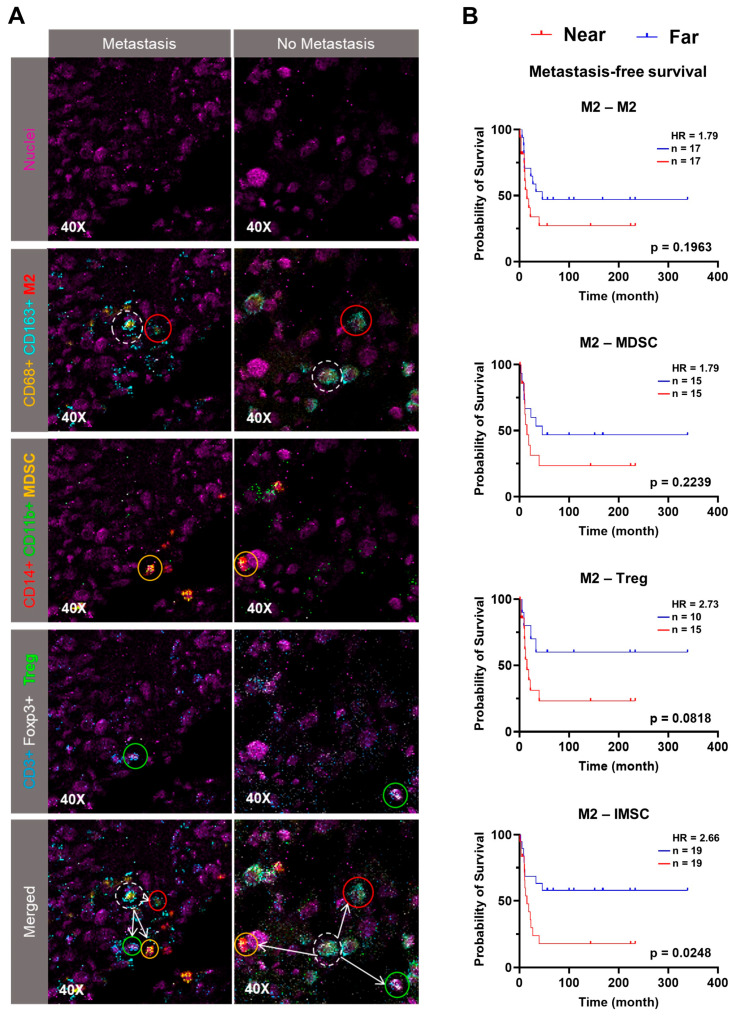
Kaplan–Meier analyses of spatial distances between immunosuppressive cell (IMSC) populations and M2 macrophages. (**A**) Representative IMC images illustrate shorter (**left**) and longer (**right**) spatial distances between M2 macrophages (M2), myeloid-derived suppressor cells (MDSC), and regulatory T cells (Treg) in metastatic versus non-metastatic patients. White dashed circle indicates a M2 cell and its neighboring M2 (red circle), MDSC (yellow circle) and Treg (green circle) cells. (**B**) Kaplan–Meier analyses evaluate metastasis-free survival based on spatial distances of M2–M2, M2–MDSC, M2–Treg, and M2–IMSC, calculated within a 250 µm radius of M2. The M2–IMSC distance per core was estimated as the mean of the median distances of M2, MDSC, and Treg from M2. Cell–cell distances were reported as the median value for each group (M2–M2: near = 0 µm, far = 34.8 µm; M2–MDSC: near = 0 µm, far = 127 µm; M2–Treg: near = 0 µm, far = 117.1 µm; M2–IMSC: near = 0 µm, far = 90.65 µm), and patients were stratified into “far” (blue) and “near” (red) groups based on the median distance of cell population from M2. A *p*-value < 0.05 was considered statistically significant.

IMC enables spatial analysis of cell populations within the OS TME, complementing the cell abundance data. We investigated the spatial distances of CD68^+^CD163^+^ M2 with themselves, Treg, and MDSC, as well as with non-immunosuppressive cell subpopulations within a 250 µm radius, the reported maximum distance for meaningful hematopoietic cell–cell communication [19]. Shorter median distances between M2–M2, M2–Treg, and M2–MDSC showed no significant correlation with MFS (Figure 4A,B). However, the collective distance of M2–IMSC was significantly associated with worse MFS (*p* = 0.0248, HR = 2.66, Figure 4B). A similar significant correlation of the distance of M2–M2 and Treg with worse MFS was also observed (*p* = 0.0301, HR = 2.64, Supplementary Figure S8). However, these spatial metrics did not correlate with RFS, indicating a preference toward distal metastasis (Supplementary Figure S9). To dissect whether this spatial relationship is specific to immunosuppressive cells, we further investigated spatial relationships between M2 and non-immunosuppressive cell populations. Shorter median distances between M2 and non-immunosuppressive cells showed no correlation with worse MFS or RFS, except for Ki-67^+^ cells (MFS: *p* = 0.0321, HR = 2.43, Supplementary Figure S10). Additionally, cell density analysis revealed no significant prognostic association between the density of any cell type surrounding M2 (Supplementary Table S4C), indicating that the prognostic impact of M2–IMSC proximity is not attributable to higher cell density. Taken together, these results suggest that the spatial organization of M2 macrophages within the OS TME, particularly their close interactions with IMSC and Ki-67^+^ tumor cells, forms a prognostically significant immunosuppressive or pro-tumor hub.

### 3.4. M2 Drives Pulmonary Metastasis in an Orthotopic Osteosarcoma Xenograft Model

**Figure 5:**
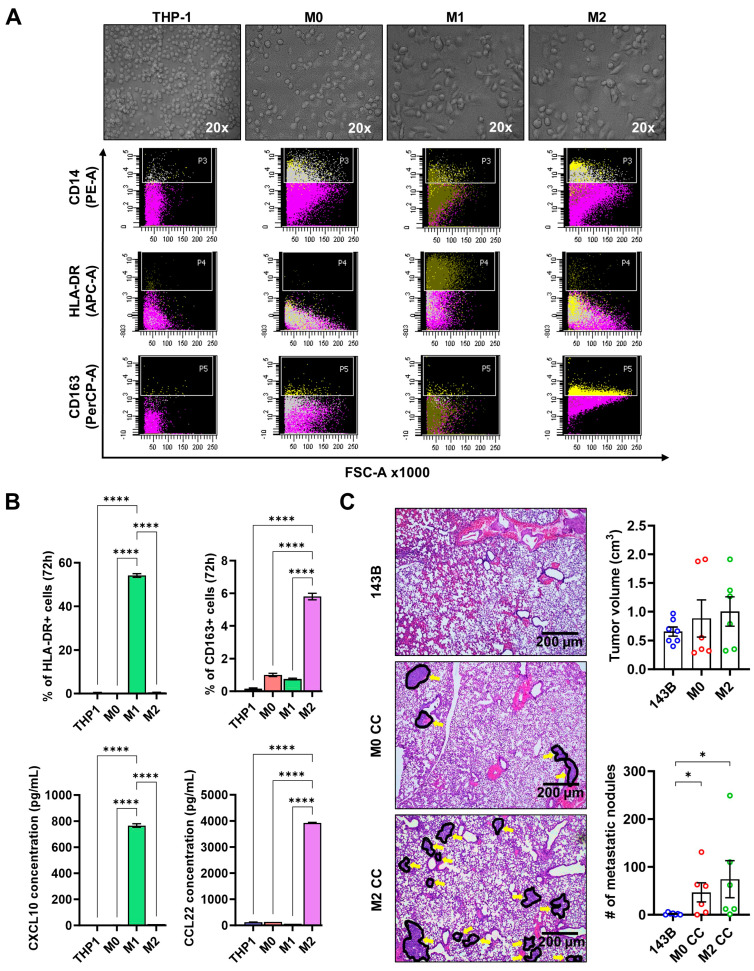
The effect of M2 macrophages on pulmonary metastasis in OS. (**A**) Upper panel: Cell morphology of THP-1-derived M0, M1, and M2 macrophages taken at 20× magnification with a light microscope. Small, round THP-1 cells treated with 10 ng/mL PMA differentiated into large, elongated M0 macrophages. M0 cells were further treated with 20 ng/mL of IFN-γ and 10 pg/mL of LPS or 20 ng/mL of IL-4 and IL-13 to induce M1 and M2 macrophage differentiation, respectively, resulting in larger, stellate-shaped cells with increased cytoplasmic-to-nuclear volume. Lower panel: Flow cytometry analysis of CD14, HLA-DR (M1 marker), and CD163 (M2 marker) expression levels in differentiated or polarized macrophages. (**B**) Confirmation of THP-1 differentiation and polarization of M0 into M1 and M2 using canonical markers specific for macrophage subtypes. Upper panel: Flow cytometry analysis of marker expression. Lower panel: ELISA analysis of conditioned media collected after 72 h of rest in macrophage maintenance medium (MMM). (**C**) (**Left**): Representative H&E-stained images of metastatic nodules in resected lungs from orthotopic xenograft mouse models intratibially injected with 143B cells alone, 143B with M0 macrophages (M0 CC), or 143B with M2 macrophages (M2 CC). Metastatic nodules are indicated with yellow arrows. (**Right**): Comparisons of primary tumor volumes calculated as 0.5 × height × width^2^ (upper) and the number of metastatic nodules in mouse models with different cell injections after 5 weeks. Each dot represents an individual mouse. Error bars indicate standard deviations, and asterisks denote statistical significance (* *p* < 0.05; **** *p* < 0.0001; unmarked = not significant).

Given the high abundance of CD68^+^CD163^+^ M2 in OS TME and their proximity to IMSC correlating with worse MFS in the IMC analysis, we evaluated their functional role in OS growth and metastasis using an orthotopic xenograft mouse model. THP-1 monocytic cells were transformed into M0 and then polarized to M1 or M2 macrophages as previously described [16]. Morphological examination showed that THP-1 cells appeared small, round, and non-adherent; PMA-differentiated M0 macrophages were large, round-to-elongated, and adherent; and M1 and M2 macrophages exhibited elongated, stellate shapes (Figure 5A, upper). Flow cytometry (Figure 5A, lower; Figure 5B, upper) and ELISA (Figure 5B, lower) confirmed M2 polarization, with elevated CD163 and CCL22 expression in M2 compared to THP-1, M0, and M1 cells, whereas M1-specific markers (HLA-DR and CXCL10) were expressed highly in M1 macrophages but absent in M2. In NOD/SCID mice, co-injection of 143B cells with M0 or M2 macrophages (3:1 ratio) into the proximal tibia did not significantly affect primary tumor growth compared to 143B alone (Figure 5C, right upper). However, M0-OS or M2-OS co-injected mice developed significantly larger and more numerous pulmonary metastatic nodules than the control (Figure 5C), indicating the metastasis-promoting effects of M0 and M2 macrophages in OS.

### 3.5. M2 Enhances Osteosarcoma Cell Migration via MIP-1α Signaling

**Figure 6:**
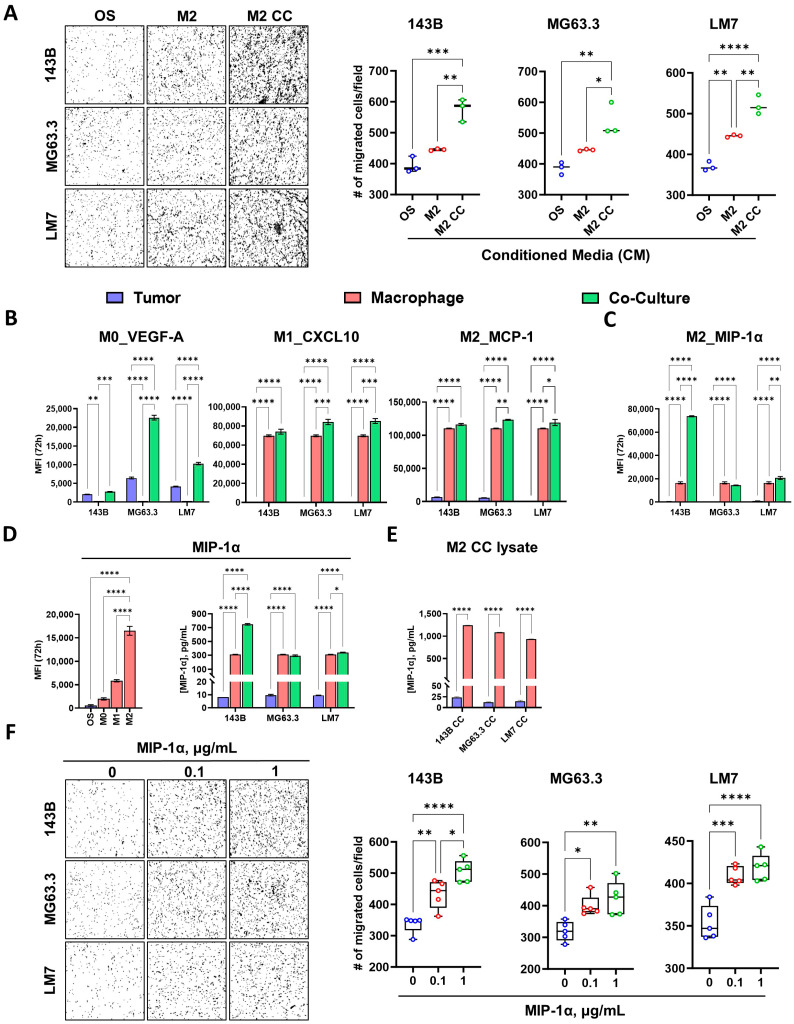
Luminex analysis of conditioned media from macrophage-OS Co-cultures and mono-cultures and the effect of MIP-1α on OS cell migration. (**A**) Left: Representative images of transwell migration assays for three OS cell lines incubated with conditioned media (CM) of 72 h OS-M2 co-cultures. OS and M2 mono-culture CM served as controls. Right: Quantification of migrated cells, calculated as the average cell count across five fields at 10× magnification. Cells were starved overnight in 0.1% FBS-supplemented DMEM before the assay. N = 3. (**B**) Differential expression of macrophage-specific markers (VEGF-A, CXCL10, and MCP-1) in non-contact co-cultures of M0, M1, and M2 macrophages with OS cell lines compared to their respective mono-cultures. A panel of 48 cytokines/chemokines in conditioned media was analyzed using the Luminex platform. (**C**) Bar plot showing significantly elevated MIP-1α levels in the CM from two of three OS-M2 co-cultures compared to mono-cultures as measured using the Luminex assay. (**D**) Left: Bar plot illustrating the significant expression of MIP-1α in M2 mono-cultures compared to M0, M1, and OS mono-cultures as measured using the Luminex assay. Right: ELISA validation of markedly increased MIP-1α levels in independent M2-OS co-cultures relative to mono-cultures. (**E**) ELISA analysis of cell lysates from M2-OS co-cultures to identify M2 macrophages as the source of MIP-1α production. (**F**) Left: Representative images of transwell migration assays for three OS cell lines treated with increasing concentrations of MIP-1α. Right: Quantification of migrated cells, calculated as the average cell count across five fields at 10× magnification. Cells were starved overnight in DMEM supplemented with 0.1% FBS before the assay. Five independent experiments were performed. Error bars represent standard deviations, and asterisks indicate statistical significance (* *p* < 0.05; ** *p* < 0.01; *** *p* < 0.001; **** *p* < 0.0001; unmarked = not significant).

To examine whether the metastatic effect of M2 is attributed to cell–cell communication between the macrophage and OS cells via soluble factors, we co-cultured three genetically diverse OS cell lines (143B, LM7, and MG63.3) with THP-1-derived M2 in a non-contact transwell system. Mono-cultures of each OS cell line and M2 served as controls. Our results revealed that the CM from the M2 co-cultures markedly promoted the tumor cell motility of 143B, MG63.3, and LM7 cells compared to the CM from OS and M2 mono-culture controls, suggesting the presence of pro-migratory soluble factors derived from M2-OS crosstalk (Figure 6A). Macrophages promote tumor progression through cytokine-mediated signaling, a key mechanism in the OS TME [20]. To identify tumor-educated M2-derived cytokines driving metastatic functions, we then measured 48 cytokines in the CM of these co- and mono-cultures using the Luminex platform. Similar co- and mono-cultures of M0 and M1 macrophages were included as additional controls. The Luminex results showed that 3, 13, and 8 differentially abundant cytokines were identified in co-cultures compared to mono-cultures for M0-OS, M1-OS, and M2-OS conditions, respectively (Supplementary Figure S11). Notably, VEGF-A (M0), CXCL10 (M1), and MCP-1 (M2), known cytokines that are expressed highly in the respective macrophage when they are educated by tumor cells, were identified (Figure 6B). Within the eight differentially abundant cytokines in M2-OS, MIP-1α (CCL3) was significantly elevated in the M2 mono-culture relative to the OS mono-culture (Figure 6C). In addition, it was significantly elevated in the M2 co-cultures with 143B (fold change = 4.3, *p* < 0.0001) and LM7 (fold change = 1.2, *p* < 0.01) cell lines compared to the M2 mono-culture control, suggesting that cytokine expression can be influenced by the presence of OS cells (Figure 6C).

MIP-1α is an interesting cytokine, as it is linked to bone resorption and osteoclast inhibition, a characteristic particularly relevant to OS [21]. Moreover, MIP-1α was significantly higher in M2 mono-culture compared to M1 and M0 mono-cultures (Figure 6D, left). Using ELISA, we validated higher MIP-1α levels in independent M2-OS co-culture samples (Figure 6D, right). Analysis of cell lysates obtained from the M2-OS co-cultures indicated significantly higher MIP-1α expression in M2 than OS cell lines (Figure 6E), corroborated by single-cell RNA-seq analysis showing elevated CCL3 expression in M2 cells marked by high expression of CD68 and CD163, but not in T cells (marked by high CD3 expression) and OS cells (marked by high STAB2 expression) in OS tumors (Supplementary Figure S12). To examine whether MIP-1α can influence the metastatic properties of OS cells, we incubated recombinant MIP-1α (0.1 and 1 µg/mL) with three OS cell lines in Boyden chambers. The result showed that the cytokine induced concentration-dependent migration of all three OS cell lines (Figure 6F), suggesting that M2-derived MIP-1α can enhance OS metastatic propensity. These findings provide a novel mechanistic insight into how M2 promotes OS metastasis via MIP-1α signaling.

## 4. Discussion

Despite advances in tumor immunology, a limited understanding of the OS TME hinders immunotherapy efficacy. Recent studies of metastatic OS lung tissues reveal an immune-cold TME with enriched PDL1-, LAG3-, and TIM3-expressing tumor-infiltrating lymphocytes, M2, and MDSC at the tumor periphery associated with poorer survival [13,14,22]. These findings indicate co-operative immune exclusion and suppression mechanisms in OS, driving adverse outcomes. Using multiplexed IMC, we characterized the composition and spatial organization of TME cell populations in 51 primary OS tumors in the tissue microarray. Compared to immunofluorescence and confocal microscopy with spectral analysis, imaging mass cytometry (IMC) enables the simultaneous detection of a substantially larger panel of markers without the limitations imposed by spectral overlap. The IMC workflow (Supplementary Figure S2) involves tissue staining with metal-conjugated antibodies, followed by laser ablation to ionize these metals and mass spectrometry to identify the markers, generating rich and multiplexed spatial data for downstream analysis. Our study focuses on tumor-infiltrating immune cells and tumor-educated stromal cells in the tumor cores. Although immunohistochemistry targeting CD163^+^ M2 would provide a simpler approach to assess abundance, we opted for multiplexed IMC to thoroughly characterize the entire TME landscape and facilitate the intricate spatial and correlation analyses that underpin our key findings on identifying prognostically significant cell populations. Over 50% of immune cells were CD68^+^ macrophages, predominantly CD68^+^HLA-DR^−^CD163^−^ M0 and CD68^+^CD163^+^ M2 subtypes over the CD68^+^HLA-DR^+^ M1 subtype, suggesting a pro-tumor and immunosuppressive TME. We report, for the first time, the prognostic significance of IMSC (M2, MDSC, and Treg) abundance and spatial proximity in OS, with higher abundance or closer IMSC-to-M2 proximity correlating with worse patient outcomes. In an orthotopic xenograft mouse model, THP-1-derived M0 or M2 macrophages promoted pulmonary metastasis. Multiplex cytokine analysis of CM from OS-M2 co-culture and mono-cultures identified MIP-1α as a novel differentially regulated M2-derived cytokine that can promote metastatic propensity in OS.

OS TME is composed of complex and heterogeneous cell populations [11,23]. Our multiplexed IMC analysis constructed a detailed map of various cell populations in OS TME, revealing a low to moderate abundance of Pan-CK^+^ and E-cadherin^+^ cells, and a high abundance of αSMA^+^, CD31^+^, Ki-67^+^, CD68^+^, and CD163^+^ cells. M0 macrophages and T cells are the dominant myeloid and lymphoid populations, respectively. As a mesenchymal tumor, OS rarely expresses epithelial markers like Pan-CK and E-cadherin. Cells exhibiting epithelial features in OS may have undergone epithelial-to-mesenchymal transition (EMT), acquiring both epithelial and mesenchymal characteristics, a process observed in aggressive sarcomas [24]. Alternatively, these features could result from aberrant marker expression or cross-reactivity with intermediate filaments, such as desmin [25]. High abundance of αSMA^+^ cells suggests the presence of abundant cancer-associated fibroblasts (CAFs) and pericytes, which can promote extracellular matrix remodeling, angiogenesis, and tumor progression [26]. CAFs are often associated with tumor progression and drug resistance [27]. CD31 is a marker of endothelial cells. A high abundance of endothelial cells indicates increased vascularity, supporting the aggressive and metastatic nature of OS [18,28]. High Ki-67 expression often correlates with worse outcomes in cancer, particularly in non-metastatic disease [29,30]. Our data showed a homogeneous high abundance of Ki-67^+^ cells in the OS cases, which is consistent with the high proliferative nature of OS.

Bone sarcomas exhibit variable immune cell infiltration, with conventional high-grade OS showing moderate-to-heavy infiltration compared to chondrosarcoma, chordoma, and Ewing sarcoma [31,32]. Prior studies, including Inagaki et al., identified CD68^+^CD14^+^ macrophages and CD3^+^ T lymphocytes as dominant components of the OS TME [11,33,34]. Similarly, Deng et al., using the CIBERSORT algorithm, reported elevated M0 and M2 macrophage ratios in OS tissues from the TARGET cohort [35]. Our analysis confirmed a moderate immune infiltrate (1–20% of cells) in OS, with M0 and M2 macrophages significantly higher than other myeloid subpopulations. T cells, particularly memory helper and cytotoxic subtypes, were more abundant than NK cells, though their predominance may reflect longevity rather than a direct role in tumor progression [36,37]. The high M0 and M2 abundance, coupled with relatively low T cell presence, indicates a pro-tumor and immunosuppressive TME in OS. OS may recruit and differentiate circulating monocytes into M0 macrophages, which are then polarized into pro-tumor M2. Work by Zhou et al., Deng et al., and others supported this notion [35,38,39]. The former study further showed that M2 was found to be enriched in OS tissues and enhanced the initiation and stemness of murine K7M2 cells in vivo [39].

The prognostic role of M2 in OS has been controversial [40]. Gomez-Brouchet et al. linked high CD163^+^ tumor-associated macrophage (TAM) infiltration to improved overall and metastasis-free survival in OS, possibly due to tissue remodeling or antitumor roles [41], while Su et al. associated high CD68^+^CCL18^+^ M2 with lung metastasis and poor prognosis in OS, indicating a pro-tumorigenic subset [42]. Besides OS, in certain lymphomas and prostate cancers, high expression correlates with better outcomes [43,44]. In adenoid cystic carcinoma, abundant M2-like TAMs in lung metastases diminished MFS despite robust immune infiltration [45]. Similarly, single-cell analysis of breast cancer showed T lymphocyte predominance in primary tumors but M2 dominance in brain metastases, implicating M2-TAMs in metastatic progression [46]. In this study, survival analysis found no significant correlation between M2 alone and worse outcomes. These discrepancies suggest that M2 alone may not be sufficient to consistently correlate with prognosis. Other immune features, e.g., low cytotoxic T cells, or the presence of other IMSC, e.g., MDSC and Treg, may also play a crucial role in outcome prediction.

Previous studies showed that high IMSC infiltration correlates with reduced dendritic cell (DC), NK cell, and effector T cell function, driving metastasis and poorer survival in OS [22,47,48]. In this study, we found that IMSC abundance inversely correlates with DC but positively associates with T cells. Our analysis revealed that the individual abundance of IMSC had no significant impact on survival, whereas their collective abundance was prognostically significant. Specifically, the collective abundance of M2 and MDSC significantly predicted worse MFS and RFS, highlighting their critical role in immunosuppression and poor prognosis. Additionally, CD44^+^ cancer stem cell abundance was associated with worse RFS, but not MFS. The role of cancer stem cells in recurrence has been well documented. For instance, enrichment of CD44^+^ cancer stem cells is linked to therapy resistance, higher recurrence rates, and shorter overall survival in breast and ovarian cancers [49,50,51]. In OS, silencing CD44^+^ cells enhances doxorubicin sensitivity and reduces malignant progression, emphasizing their role in driving recurrence in aggressive tumors like OS [52,53]. Consistent with our finding that the abundance of individual TME cell populations does not significantly predict outcomes, we performed an additional analysis comparing the abundance of various cell populations between patients with and without metastasis, as well as between those with and without recurrence. A Mann–Whitney U test revealed no significant differences in the abundance of any single cell population across these groups (Supplementary Figure S13), further supporting the conclusion that individual cell populations alone are not predictive of clinical outcomes.

Besides cell abundance, we investigated whether the spatial organization of IMSC has any association with OS outcome by performing a spatial analysis of IMSC with M2, the most abundant immunosuppressive cell population. Our result demonstrated that patients with closer M2–IMSC proximity (M2–IMSC proximal) exhibited a significantly higher metastasis risk compared to those with distant IMSC (M2–IMSC distal), with a trend toward worse recurrence-free survival. The collective proximity of M2, MDSC, and Treg outperformed other paired combinations, e.g., M2–Treg, in predicting metastasis-free survival. While the collective abundance of M2 and Treg did not correlate with clinical outcomes, the shorter distance of M2–M2 and Treg was a predictor of worse MFS (*p* = 0.0301, HR = 2.64, Supplementary Figure S8), but not RFS. This finding suggests that the spatial proximity between M2 and Treg, involving direct cell–cell contact or short-range signaling, is a critical factor driving metastatic progression, even if their combined cell abundance is not. While a prior study from Lacinski et al. has indicated the prognostic significance of spatial distances among IMSC, effector immune cells, and OS cells, our findings indicate that the spatial organization of M2 with IMSC also correlates with OS prognosis [14].

Furthermore, our study revealed that shorter median distances between M2 and Ki-67^+^ cells were associated with worse MFS, but not RFS. Ki-67, a marker of cell proliferation, is well-established in the tumor microenvironment (TME) as an indicator of actively dividing cells, likely representing tumor cells in our analysis. The proximity of M2 to OS cells may promote tumor growth, survival, and metastatic spread. The specific correlation with MFS suggests that the distance between M2 and OS cells is critical for the establishment and progression of distant metastases, which is consistent with our mouse study showing the ability of M2 to promote pulmonary metastasis. Notably, our spatial density analysis of TME cells relative to M2 showed no significant correlation with MFS or RFS, showing that cell density does not significantly influence clinical outcomes. This indicates that the prognostic significance of the distance between M2 macrophages and immunosuppressive cells is not solely a function of cell density. Taken together, our findings suggest that M2 macrophages, IMSC, and Ki-67^+^ tumor cells form an immunosuppressive and pro-tumor functional hub that contributes to adverse clinical outcomes.

Our result is also supported by a previous study showing spatial clustering of M2, MDSC, and Treg near tumor cells forms immunosuppressive hubs, which were observed in OS pulmonary metastases with peritumoral macrophage accumulation and intratumoral lymphocyte exclusion [13]. These hubs likely create barriers, limiting T-cell infiltration and cytotoxic activity, a mechanism seen in other poor prognostic cancers [54,55]. IMSC may also inhibit DC maturation, impairing T cell priming without reducing T cell numbers [56,57]. In OS pulmonary metastases, peripheral MDSC accumulation and M2 dominance with lymphocyte exclusion at the tumor interface underscore co-operative immunosuppression [13]. Similar patterns in melanoma and colorectal cancer show that effector T cell proximity to tumors enhances killing, while immunosuppressive cell clustering impairs immunity [58,59,60]. In non-small cell lung cancer, shorter M2-M1 distances correlate with poor outcomes, and in Hodgkin lymphoma, Treg are closer to M2 than MDSC, suggesting unique interactions [61,62]. Our results suggest that spatial immune architecture drives anti-tumor immune efficacy and survival in OS. Development of a means to disrupt the immunosuppressive hub formed by IMSC may create a new immunotherapy approach for OS.

M2-mediated tumor growth and metastasis are well-documented across cancers. For instance, Su et al. showed that M2-derived CCL18 enhances OS metastasis [42]. In addition, M2 inhibition with all-trans retinoic acid reduces OS initiation and stemness [39]. Mechanistically, M2 enhances tumor survival via pathways like CCL2/PI3K/Akt/mTOR in breast cancer, IL-6/STAT3 in colon and pancreatic cancers, and CHI3L1 in gastric/breast cancers [63,64,65,66]. In our study, we co-injected 143B with THP-1-derived M2 orthotopically, demonstrating their promotion of pulmonary metastasis in a mouse model. This aligns with Tatsuno et al., who showed similar effects using CM from OS-M2 co-cultures [67]. In contrast to their study, our co-injection system enables continuous tumor–macrophage crosstalk, which better mimics the physiological conditions of how M2 and OS tumor cells interact with each other. In addition to M2, we found that M0 macrophages also promoted lung metastases, suggesting that the plasticity of M0 macrophages may enable polarization to pro-metastatic M2-like phenotypes during the interaction with OS cells at the tumor site. This result is supported by our data showing M0 macrophages of the M0-OS co-cultures had a significantly higher M2 marker (CD163) expression than M1 marker (HLA-DR) expression (Supplementary Figure S14). Although immunocompetent mouse models are available, we chose to use an immunodeficient mouse model because our main goal was to study the metastatic effect of M2. In the presence of other immune cells, the M2-specific effect will be compromised and difficult to dissect. Further studies will be needed to examine the metastatic effect of M2 in the presence of other immune cells using immunocompetent mouse models. Nonetheless, our mouse findings complement the prognostic significance of M2–IMSC in the IMC analysis by demonstrating the causal role of M2 in driving OS metastasis.

Cytokine/chemokine-mediated crosstalk is a key communication mechanism in the TME [20]. M2 expresses high levels of CD163, CD206, CCL18, and CCL22, releasing anti-inflammatory (TGF-β, IL-4, and IL-13) and inflammatory (IL-6, IL-12, and TNF-α) factors, as well as tumor-promoting arginase-1 and VEGF, modulated by TME signals [68]. Our Luminex analysis of CM from M2-OS co-cultures identified M2-derived MIP-1α as a differentially expressed cytokine. It was expressed highly in M2 and was induced further by co-culturing with OS cell lines, suggesting that OS cells may produce yet-to-be-identified factors that can influence the expression of MIP-1α in M2. Interestingly, this stimulatory effect was dependent on the specific OS cell line used, with the highest effect on 143B, then LM7 and MG63.3. This observation is corroborated by the metastatic propensity of the three OS cell lines, where 143B is more aggressive than the other two. In addition, our Luminex data showed that IL-1α was expressed at a higher level in 143B than in the other two OS cell lines (Supplementary Figure S15). IL-1 is a pro-inflammatory cytokine that has been known to influence MIP-1α production [69]. At the functional level, we verified the metastatic role of MIP-1α by showing that recombinant MIP-1α significantly enhanced tumor cell migration in three OS cell lines in a dose-dependent manner. While the role of MIP-1α in OS is still unclear, Liao and Tsai et al. linked the cytokine to JNK/ERK/p38-mediated angiogenesis, suggesting a metastatic role [70]. Similarly, MIP-1α promoted migration in multiple myeloma and esophageal squamous cell carcinoma via CCR5/AKT/ERK pathways [71,72]. Identification of MIP-1α provides a novel mechanistic insight into how M2 promotes OS metastasis, particularly given its established role in bone resorption, which may facilitate tumor cell dissemination and colonization in bone-rich environments. However, since our cytokine panel is limited, the use of additional panels may identify other M2-derived cytokines or soluble factors that can promote OS metastatic propensity.

## 5. Conclusions

Our study reveals the importance of TME in OS metastasis, but there are methodological limitations. The number of markers analyzed was relatively limited, though it is typical for IMC studies. With the advance of spatial profiling, additional markers can be included for analyzing other cell populations or subpopulations and cell states to expand this study. Although, to our knowledge, this is the largest IMC study in OS to date, the sample size remains insufficiently powered to fully correct for multiple testing in the survival analyses. As such, our analysis should be considered exploratory, and the prognostic significance of the identified markers requires future validation.

Despite the limitations, our study generates a detailed landscape of TME cells in OS and offers significant potential to advance OS clinical management by identifying novel prognostic biomarker candidates and therapeutic targets within the TME. Our analysis reveals the predominance of macrophages, with M0 and M2 subtypes significantly outnumbering other immune populations. For the first time, we demonstrate that the collective abundance of M2 and MDSC is significantly associated with reduced MFS and RFS, while the spatial proximity of M2 macrophages and IMSC or Ki-67^+^ cells correlates with diminished MFS. In vivo, we establish a causal role for M2 macrophages in promoting pulmonary metastasis using an orthotopic mouse model and identify M2-derived MIP-1α (CCL3) as a potential chemokine driving OS cell metastatic potential. These findings introduce candidate prognostic biomarkers for risk stratification in OS, which warrant further validation to inform tailored therapeutic strategies. The identification of MIP-1α as an M2-derived pro-metastatic mediator highlights a novel therapeutic target to suppress OS metastasis, paving the way for the development of precision immunotherapies. These insights may guide future research and clinical trials to improve outcomes in metastatic OS.

## Figures and Tables

**Table 1:** Patient demographics of the OS cases (n = 58) used in this study. The number of metastatic cases = 34.

Characteristics	N/Total (%)
Age (yrs)	
<18	24/58 (41)
≥18	30/58 (52)
Unknown	4/58 (7)
Sex	
Male	27/58 (47)
Female	4/58 (6)
Unknown	27/58 (47)
Race	
Caucasian	45/58 (78)
African American	6/58 (10)
Other	7/58 (12)
Histologic subtype	
Osteoblastic	19/58 (33)
Fibroblastic	18/58 (31)
Chondroblastic	10/58 (17)
Telangiectatic	4/58 (7)
Other	7/58 (12)
Primary tumor site	
Femur	27/58 (47)
Tibia	12/58 (21)
Other	19/58 (32)
Stage	
IIA	23/58 (40)
IIB	20/58 (34)
III or higher	6/58 (10)
Undetermined	9/58 (16)
Metastasis at diagnosis or during follow-up	
Yes	30/58 (52)
No	24/58 (41)
Unknown	4/58 (7)
Site of metastasis	
Lung	28/34 (82)
Other	6/34 (18)
Disease recurrence after resection	
Yes	30/58 (52)
No	23/58 (40)
Unknown	5/58 (8)

## Data Availability

Generated data supporting our findings are available from the corresponding author upon request.

## Supplementary Materials

The following supporting information can be downloaded at: https://www.mdpi.com/article/10.3390/cancers17172780/s1, Table S1: Detailed clinical information of individual tumor cores in the OS tissue microarray. Table S2: The sources and experimental details of the antibodies used in imaging mass cytometry. Table S3: Markers used to annotate various cell populations and subpopulations in the osteosarcoma tumor microenvironment. Table S4: Correlation of cell abundance and M2-cell distance and density with metastasis-free survival (MFS) and recurrence-free survival (RFS). Figure S1: Representative hematoxylin and eosin (H&E) stained images of all 58 tumor cores included in the tissue microarray. Figure S2: Schematic overview of the imaging mass cytometry (IMC) workflow utilized in this study. Figure S3: Quality control of tumor cores in the tissue microarray based on nuclear and marker staining quality. Figure S4: IMC images of representative tumor cores in the tissue microarray showing cells stained with the nuclei (pink) and each of the 25 markers (green/blue) that passed QC. Figure S5: Analysis of extracellular and cellular collagen 1 in tumor cores. Figure S6: Distribution of immune cell subtypes in tumor cores. Figure S7: Kaplan–Meier analysis of stem cells (StemC). Figure S8: Kaplan–Meier analysis of metastasis-free survival by spatial proximity of immune cells. Figure S9: Kaplan–Meier analysis of recurrence-free survival by spatial proximity of immune cells. Figure S10: Kaplan–Meier analysis of the spatial distance of Ki-67^+^ cells to M2. Figure S11: Luminex analysis of macrophage co-cultures and monocultures. Figure S12: UMAPs of scRNAseq data from publicly available GSE152048 and GSE162454 datasets showing the cellular expression of MIP-1α (CCL3), alongside CD68 and CD163 (M2 macrophages), CD3D (T cells), and SATB2 (OS cells). Figure S13: Cell phenotyping of OS TMA stratified into metastatic/recurrence and non-metastatic/non-recurrent patient groups. Figure S14: OS-induced expression of CD163 and HLA-DR in M0 macrophages. Figure S15: IL-1α Expression in the conditioned media (CM) of OS cell lines.

## Abbreviations

The following abbreviations are used in this manuscript:

OS	Osteosarcoma
TME	Tumor Microenvironment
M2	M2 Macrophage
MDSC	Myeloid-Derived Suppressor Cell
Treg	Regulatory T Cell
IMSC	Immunosuppressive Cell
MIP-1α/CCL3	Macrophage Inflammatory Protein-1α
R/M	Relapsed or Metastatic
IMC	Imaging Mass Cytometry
TOF	Time-of-Flight
CM	Conditioned Media
MFI	Median Fluorescence Intensities
RFS	Recurrence-Free Survival
MFS	Metastasis-Free Survival
HR	Hazard Ratio
cCOL1	Collagen Type 1 (Cellular)
eCOL1	Collagen Type 1 (Non-cellular)
cNK	Classical NK Cell
aNK	Activated NK Cell
tNK	T-like NK Cell
Mono	Monocyte
DC	Dendritic Cell
hT	Helper T Cell
cT	Cytotoxic T Cell
mhT	Memory T Helper Cell
mcT	Memory Cytotoxic T Cell
tfhT	T Follicular Helper T Cell
ahT	Activated Helper T Cell
ecT	Effector Cytotoxic T Cell
acT	Activated Cytotoxic T Cell
nhT	Naïve Helper T Cell
ncT	Naïve Cytotoxic T Cell
Endo	Endothelial Cell
Epi-like	Epithelial-like Cell
Fib	Fibroblast
StemC	Stem Cell
TAM	Tumor-Associated Macrophage
