# Supplementary material for: Multiplex Imaging Mass Cytometry Reveals Prognostic Immunosuppressive Subpopulations and Macrophage-Driven Metastasis in Osteosarcoma
Source: Cancers (Basel). 2025 Aug 26;17(17):2780. doi: 10.3390/cancers17172780 (PMC12427482; doi:10.3390/cancers17172780)
Supplement: Supplementary file 1 [file cancers-17-02780-s001.zip › Supplementary Figures_IMC_Gyau et al 2025.v10.pptx]

## Slide 1
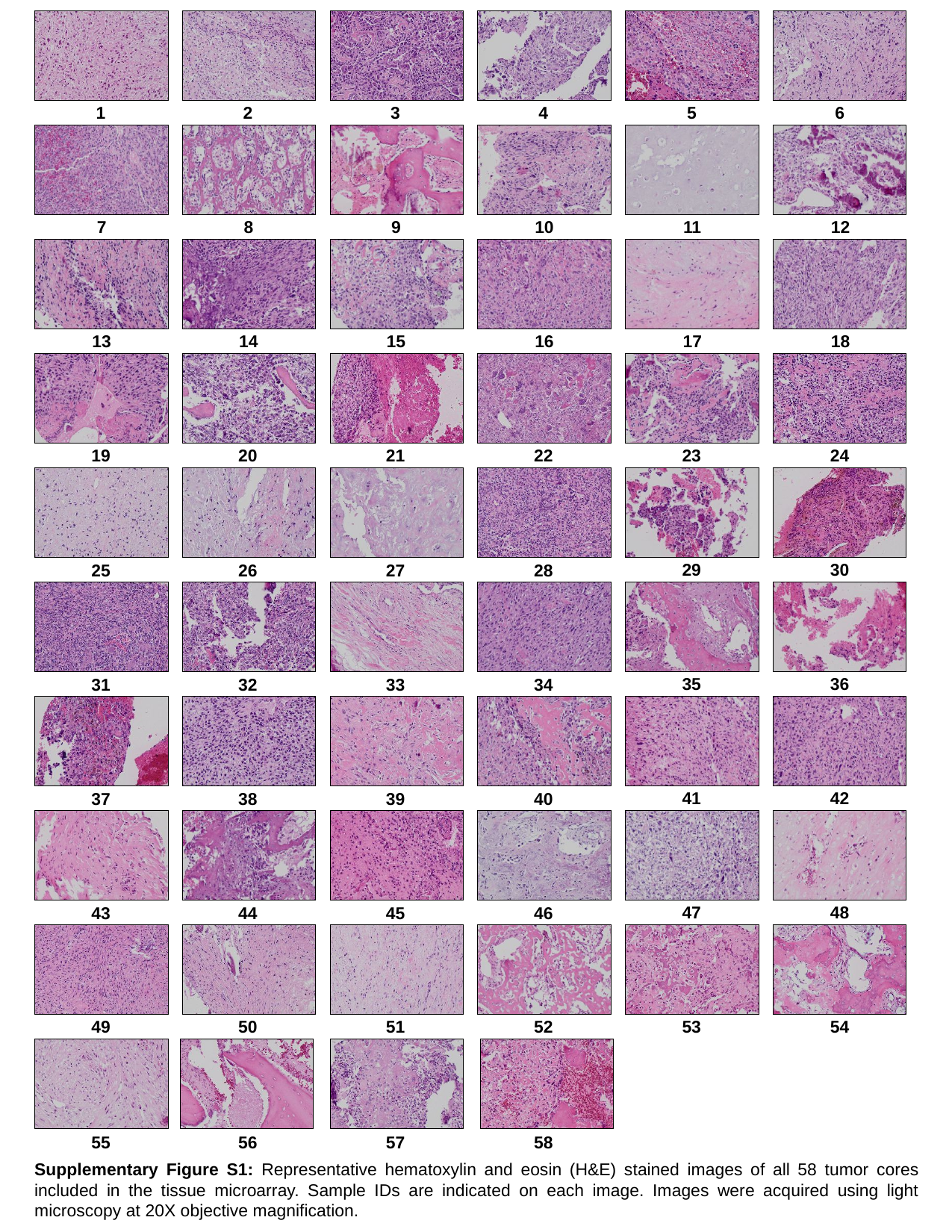

1
2
3
4
5
6
11
12
7
8
9
10
17
18
13
14
15
16
23
24
19
20
21
22
29
30
25
26
27
28
35
36
31
32
33
34
41
42
37
38
39
40
47
48
43
44
45
46
49
50
51
52
53
54
55
56
57
58
Supplementary Figure S1: Representative hematoxylin and eosin (H&E) stained images of all 58 tumor cores included in the tissue microarray. Sample IDs are indicated on each image. Images were acquired using light microscopy at 20X objective magnification.

## Slide 2
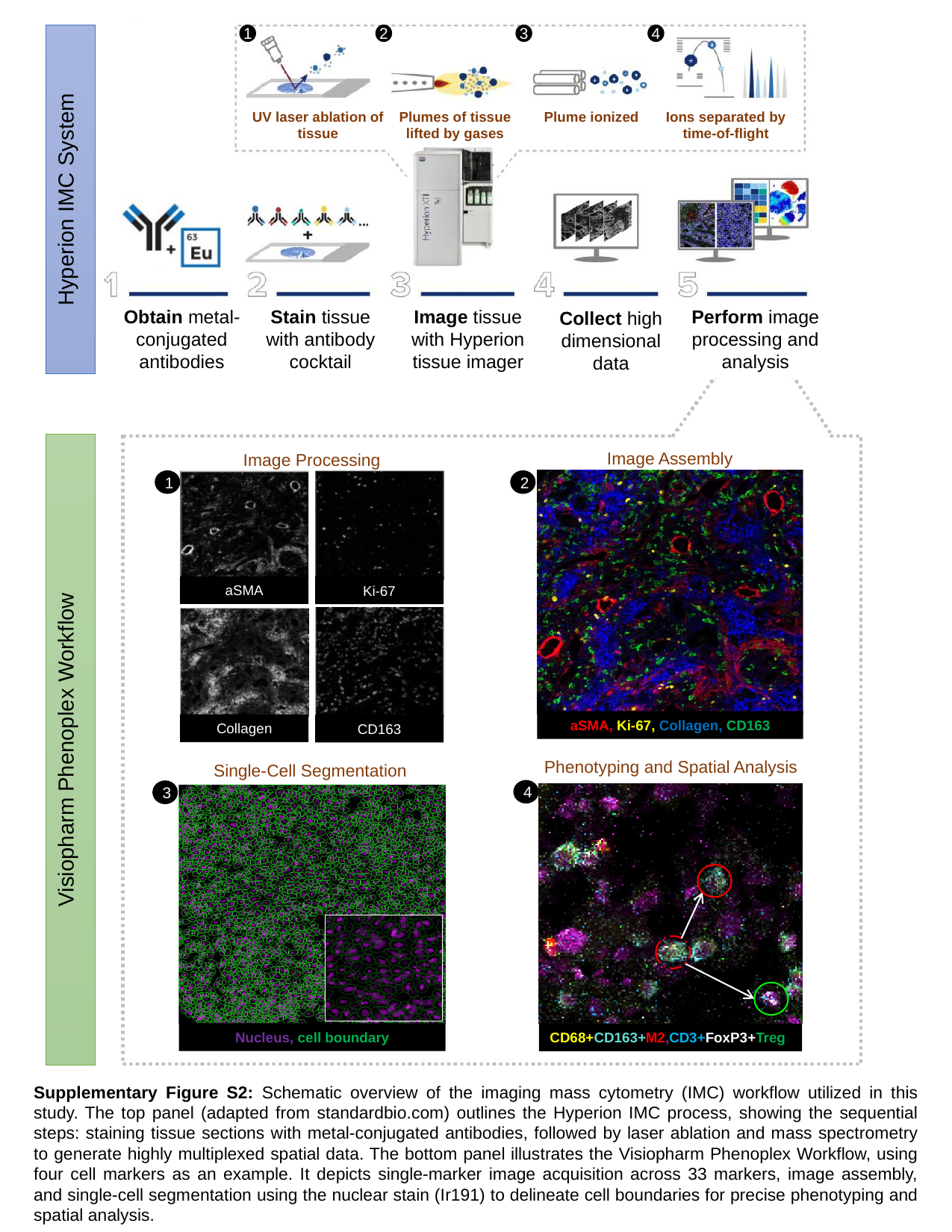

Hyperion IMC System
1
2
3
4
UV laser ablation of tissue
Plumes of tissue lifted by gases
Plume ionized
Ions separated by time-of-flight
Obtain metal-conjugated antibodies
Stain tissue with antibody cocktail
Image tissue with Hyperion tissue imager
Perform image processing and analysis
Collect high dimensional data
Visiopharm Phenoplex Workflow
Image Assembly
Image Processing
aSMA
Ki-67
Collagen
CD163
1
2
aSMA, Ki-67, Collagen, CD163
Phenotyping and Spatial Analysis
4
CD68+CD163+M2,CD3+FoxP3+Treg
Single-Cell Segmentation
3
Nucleus, cell boundary
Supplementary Figure S2: Schematic overview of the imaging mass cytometry (IMC) workflow utilized in this study. The top panel (adapted from standardbio.com) outlines the Hyperion IMC process, showing the sequential steps: staining tissue sections with metal-conjugated antibodies, followed by laser ablation and mass spectrometry to generate highly multiplexed spatial data. The bottom panel illustrates the Visiopharm Phenoplex Workflow, using four cell markers as an example. It depicts single-marker image acquisition across 33 markers, image assembly, and single-cell segmentation using the nuclear stain (Ir191) to delineate cell boundaries for precise phenotyping and spatial analysis.

## Slide 3
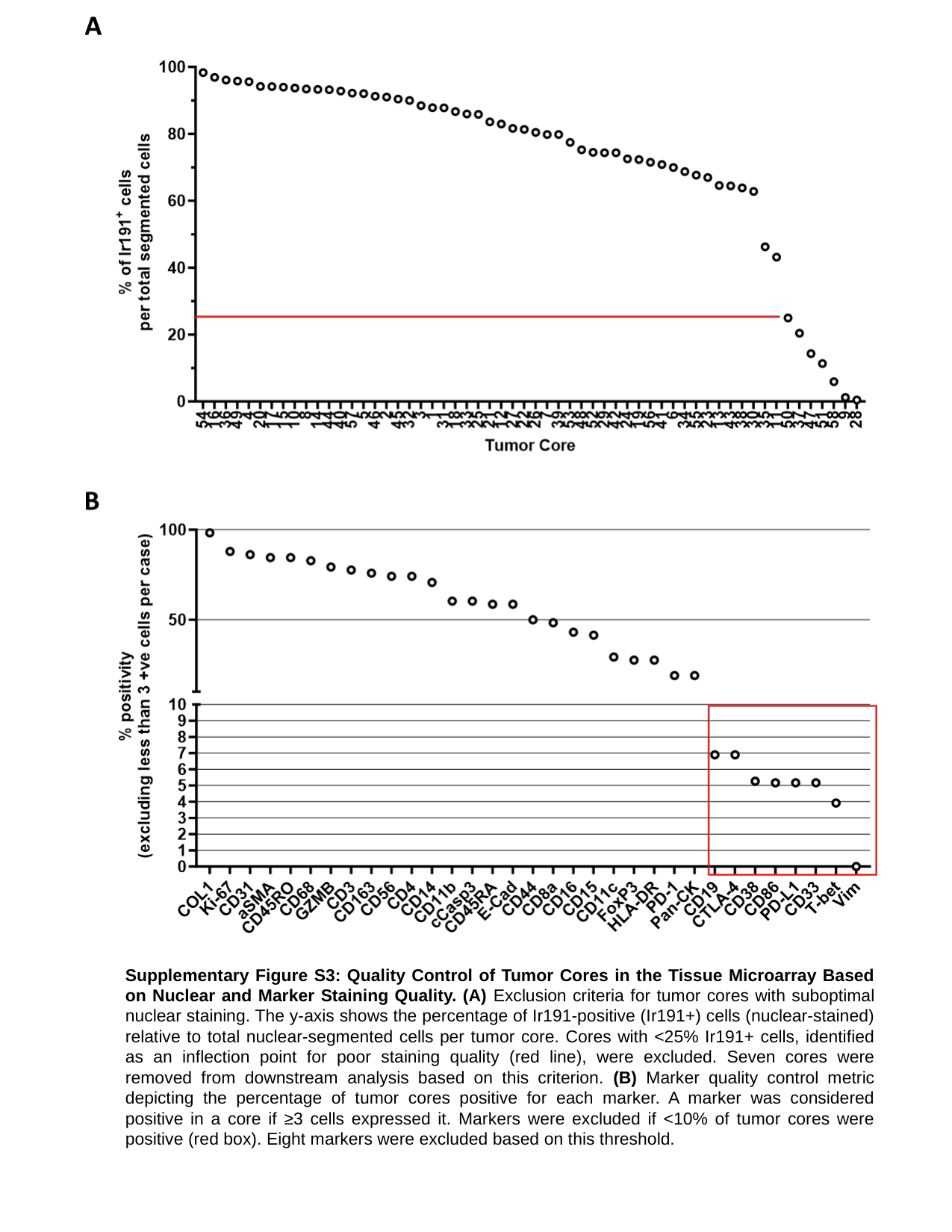

Supplementary Figure S3: Quality Control of Tumor Cores in the Tissue Microarray Based on Nuclear and Marker Staining Quality. (A) Exclusion criteria for tumor cores with suboptimal nuclear staining. The y-axis shows the percentage of Ir191-positive (Ir191+) cells (nuclear-stained) relative to total nuclear-segmented cells per tumor core. Cores with <25% Ir191+ cells, identified as an inflection point for poor staining quality (red line), were excluded. Seven cores were removed from downstream analysis based on this criterion. (B) Marker quality control metric depicting the percentage of tumor cores positive for each marker. A marker was considered positive in a core if ≥3 cells expressed it. Markers were excluded if <10% of tumor cores were positive (red box). Eight markers were excluded based on this threshold.

## Slide 4
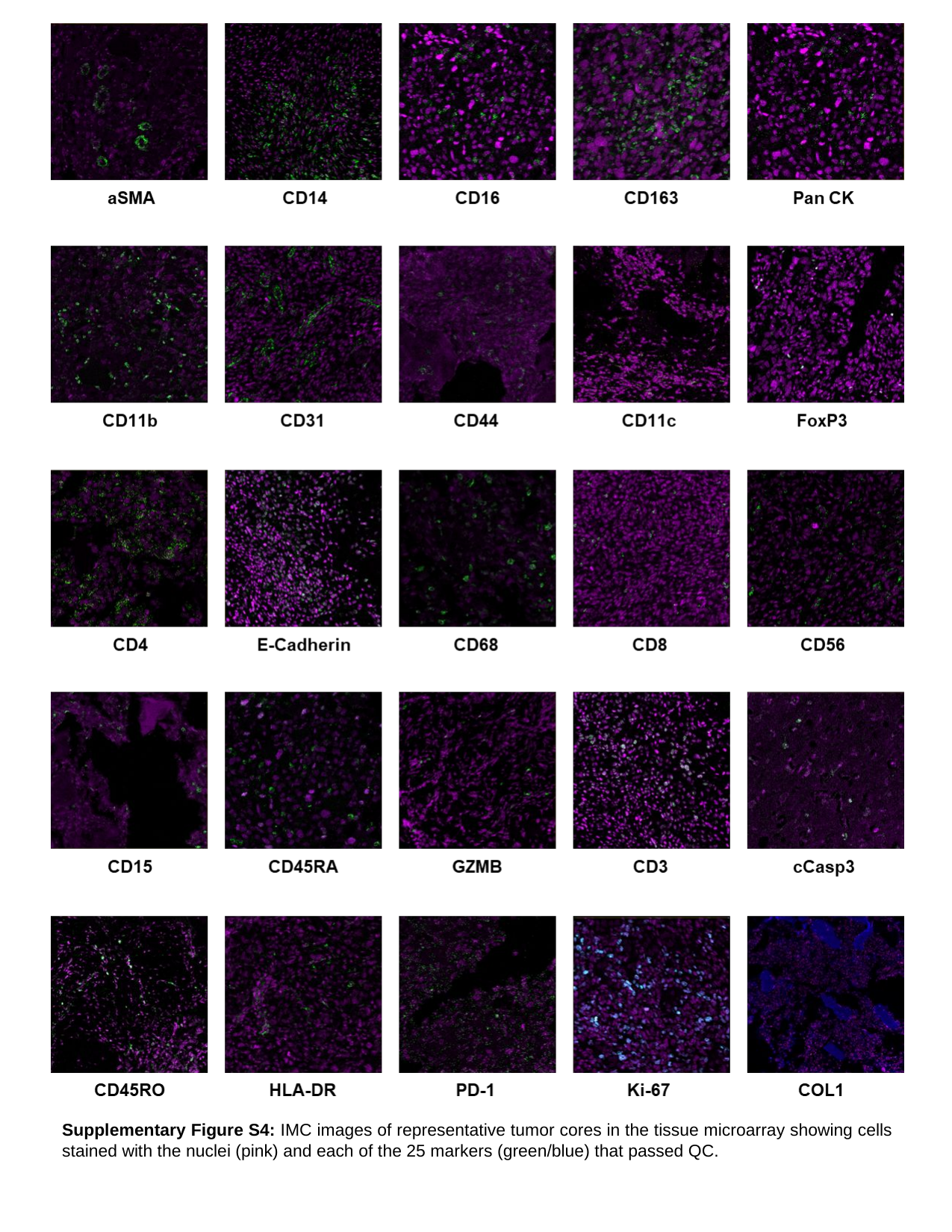

Supplementary Figure S4: IMC images of representative tumor cores in the tissue microarray showing cells stained with the nuclei (pink) and each of the 25 markers (green/blue) that passed QC.

## Slide 5
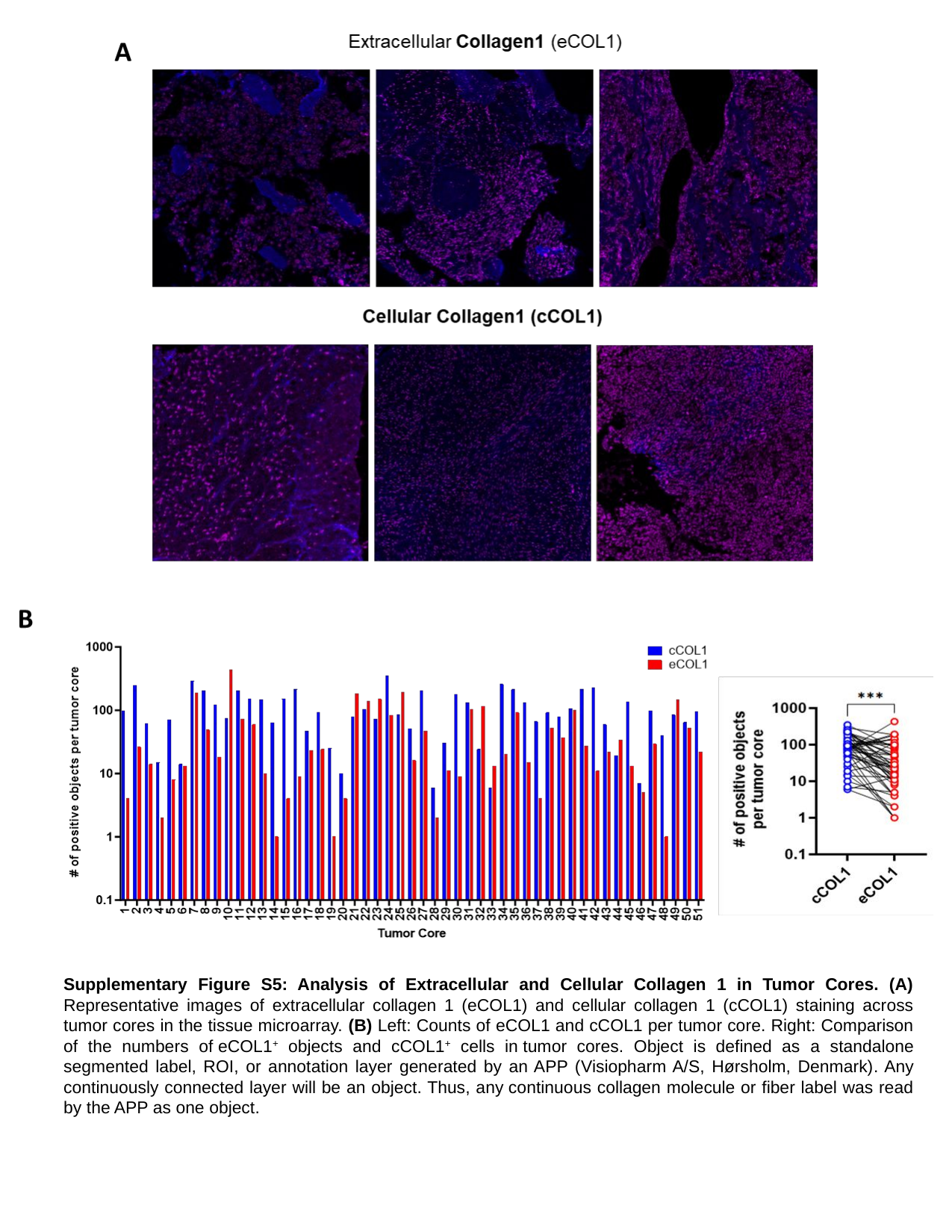

Supplementary Figure S5: Analysis of Extracellular and Cellular Collagen 1 in Tumor Cores. (A) Representative images of extracellular collagen 1 (eCOL1) and cellular collagen 1 (cCOL1) staining across tumor cores in the tissue microarray. (B) Left: Counts of eCOL1 and cCOL1 per tumor core. Right: Comparison of the numbers of eCOL1+ objects and cCOL1+ cells in tumor cores. Object is defined as a standalone segmented label, ROI, or annotation layer generated by an APP (Visiopharm A/S, Hørsholm, Denmark). Any continuously connected layer will be an object. Thus, any continuous collagen molecule or fiber label was read by the APP as one object.

## Slide 6
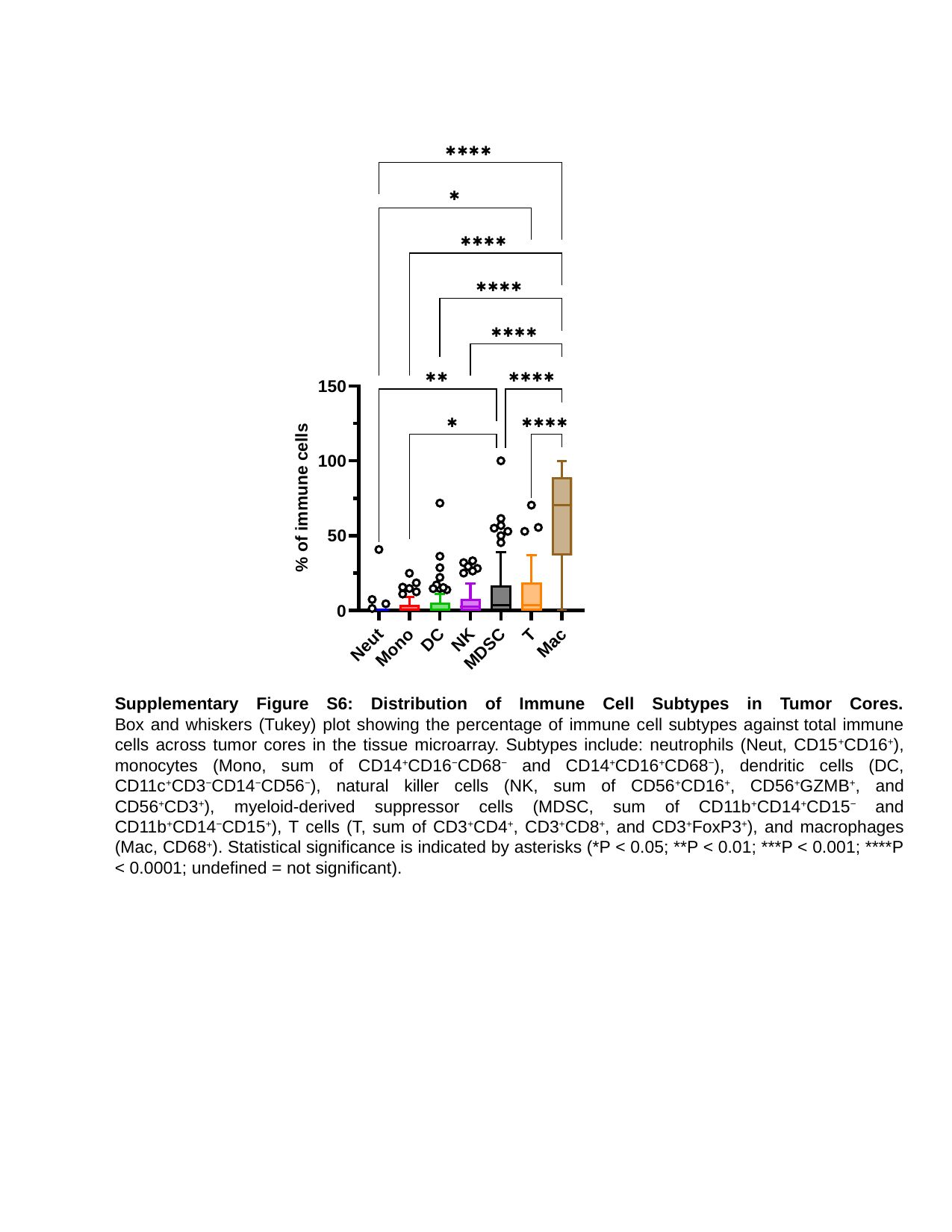

Supplementary Figure S6: Distribution of Immune Cell Subtypes in Tumor Cores.
Box and whiskers (Tukey) plot showing the percentage of immune cell subtypes against total immune cells across tumor cores in the tissue microarray. Subtypes include: neutrophils (Neut, CD15+CD16+), monocytes (Mono, sum of CD14+CD16−CD68− and CD14+CD16+CD68−), dendritic cells (DC, CD11c+CD3−CD14−CD56−), natural killer cells (NK, sum of CD56+CD16+, CD56+GZMB+, and CD56+CD3+), myeloid-derived suppressor cells (MDSC, sum of CD11b+CD14+CD15− and CD11b+CD14−CD15+), T cells (T, sum of CD3+CD4+, CD3+CD8+, and CD3+FoxP3+), and macrophages (Mac, CD68+). Statistical significance is indicated by asterisks (*P < 0.05; **P < 0.01; ***P < 0.001; ****P < 0.0001; undefined = not significant).

## Slide 7
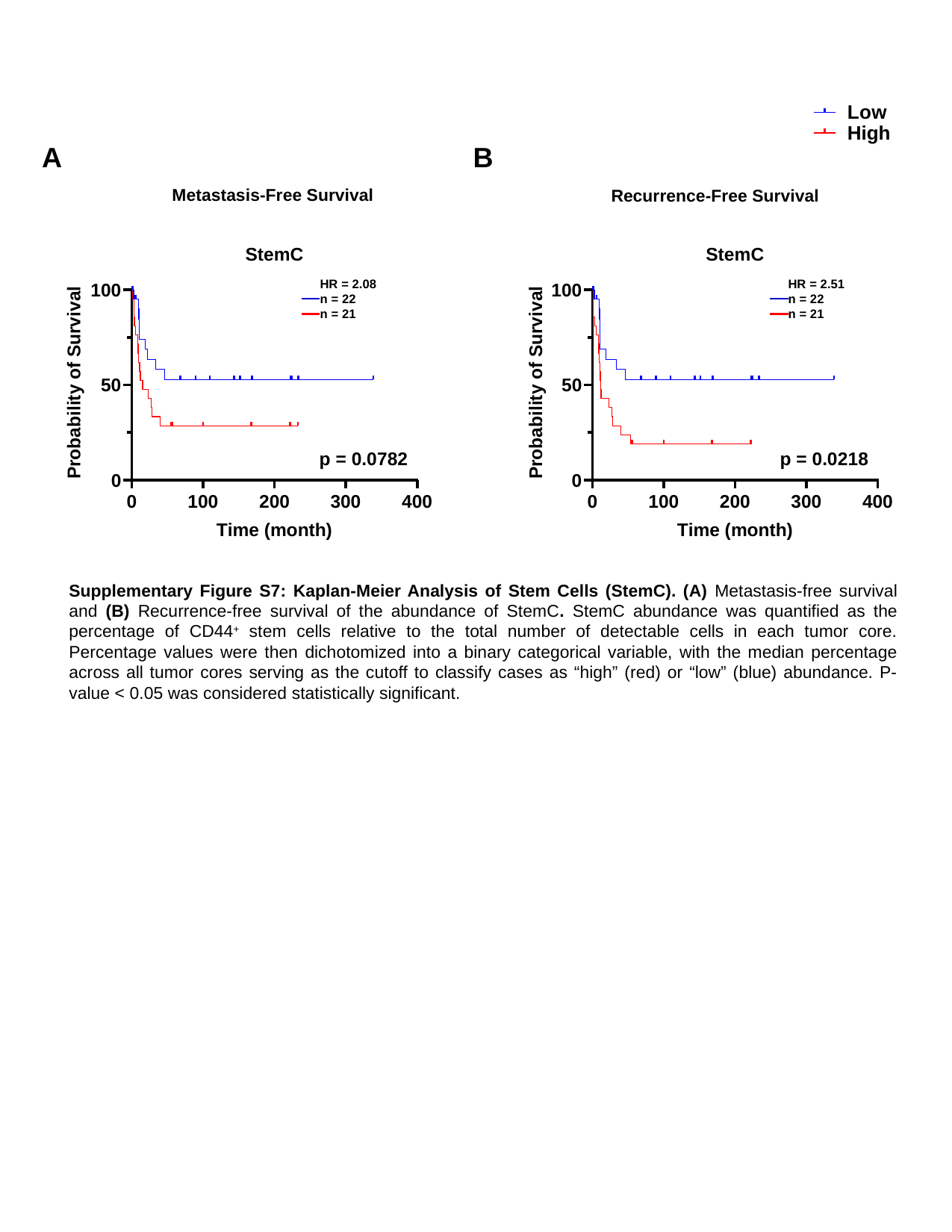

A
B
Metastasis-Free Survival
Recurrence-Free Survival
HR = 2.08
n = 22
n = 21
HR = 2.51
n = 22
n = 21
Supplementary Figure S7: Kaplan-Meier Analysis of Stem Cells (StemC). (A) Metastasis-free survival and (B) Recurrence-free survival of the abundance of StemC. StemC abundance was quantified as the percentage of CD44+ stem cells relative to the total number of detectable cells in each tumor core. Percentage values were then dichotomized into a binary categorical variable, with the median percentage across all tumor cores serving as the cutoff to classify cases as “high” (red) or “low” (blue) abundance. P-value < 0.05 was considered statistically significant.

## Slide 8
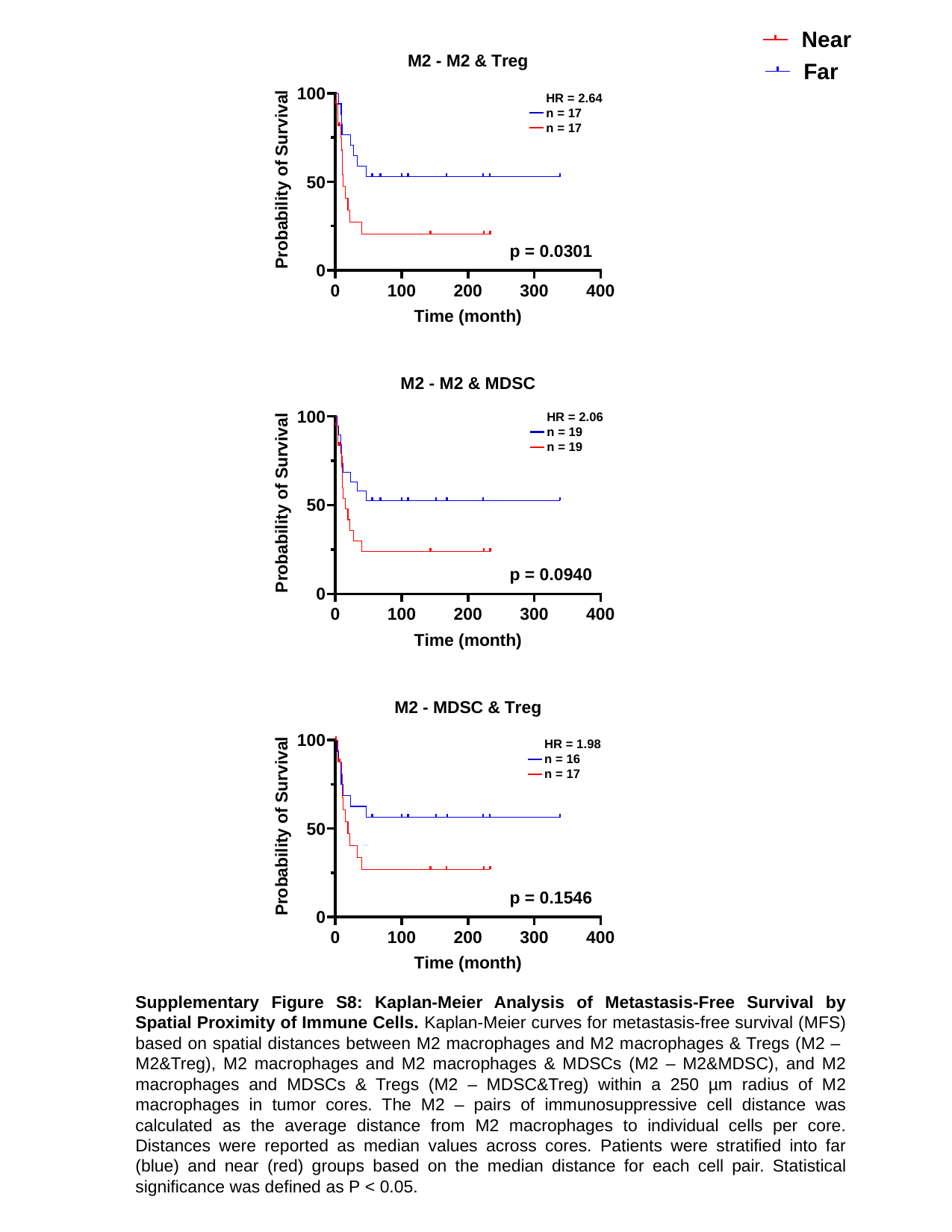

HR = 2.64
n = 17
n = 17
HR = 2.06
n = 19
n = 19
HR = 1.98
n = 16
n = 17
Supplementary Figure S8: Kaplan-Meier Analysis of Metastasis-Free Survival by Spatial Proximity of Immune Cells. Kaplan-Meier curves for metastasis-free survival (MFS) based on spatial distances between M2 macrophages and M2 macrophages & Tregs (M2 – M2&Treg), M2 macrophages and M2 macrophages & MDSCs (M2 – M2&MDSC), and M2 macrophages and MDSCs & Tregs (M2 – MDSC&Treg) within a 250 µm radius of M2 macrophages in tumor cores. The M2 – pairs of immunosuppressive cell distance was calculated as the average distance from M2 macrophages to individual cells per core. Distances were reported as median values across cores. Patients were stratified into far (blue) and near (red) groups based on the median distance for each cell pair. Statistical significance was defined as P < 0.05.

## Slide 9
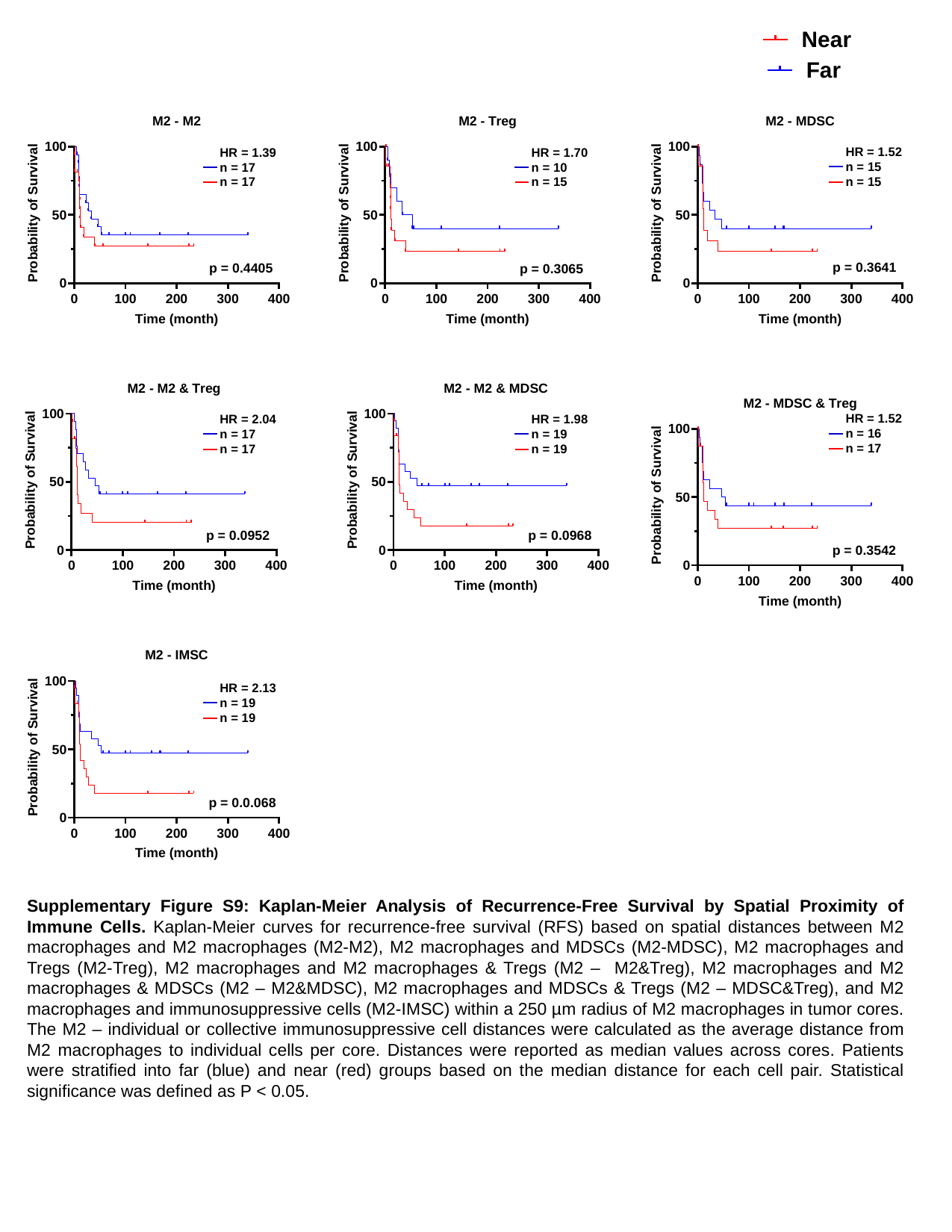

HR = 1.52
n = 15
n = 15
HR = 1.39
n = 17
n = 17
HR = 1.70
n = 10
n = 15
HR = 1.52
n = 16
n = 17
HR = 2.04
n = 17
n = 17
HR = 1.98
n = 19
n = 19
HR = 2.13
n = 19
n = 19
Supplementary Figure S9: Kaplan-Meier Analysis of Recurrence-Free Survival by Spatial Proximity of Immune Cells. Kaplan-Meier curves for recurrence-free survival (RFS) based on spatial distances between M2 macrophages and M2 macrophages (M2-M2), M2 macrophages and MDSCs (M2-MDSC), M2 macrophages and Tregs (M2-Treg), M2 macrophages and M2 macrophages & Tregs (M2 – M2&Treg), M2 macrophages and M2 macrophages & MDSCs (M2 – M2&MDSC), M2 macrophages and MDSCs & Tregs (M2 – MDSC&Treg), and M2 macrophages and immunosuppressive cells (M2-IMSC) within a 250 µm radius of M2 macrophages in tumor cores. The M2 – individual or collective immunosuppressive cell distances were calculated as the average distance from M2 macrophages to individual cells per core. Distances were reported as median values across cores. Patients were stratified into far (blue) and near (red) groups based on the median distance for each cell pair. Statistical significance was defined as P < 0.05.

## Slide 10
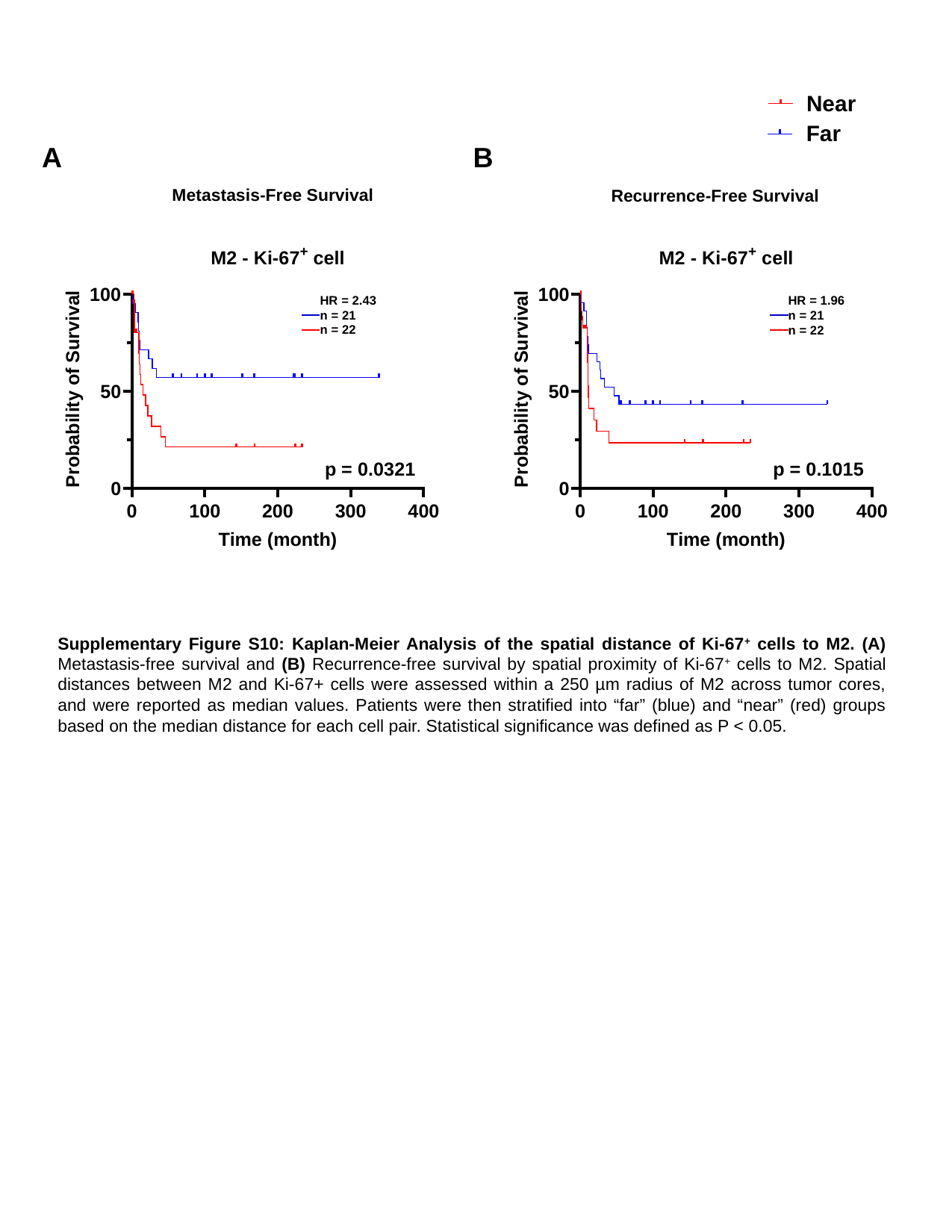

A
B
Metastasis-Free Survival
Recurrence-Free Survival
HR = 2.43
n = 21
n = 22
HR = 1.96
n = 21
n = 22
Supplementary Figure S10: Kaplan-Meier Analysis of the spatial distance of Ki-67+ cells to M2. (A) Metastasis-free survival and (B) Recurrence-free survival by spatial proximity of Ki-67+ cells to M2. Spatial distances between M2 and Ki-67+ cells were assessed within a 250 µm radius of M2 across tumor cores, and were reported as median values. Patients were then stratified into “far” (blue) and “near” (red) groups based on the median distance for each cell pair. Statistical significance was defined as P < 0.05.

## Slide 11
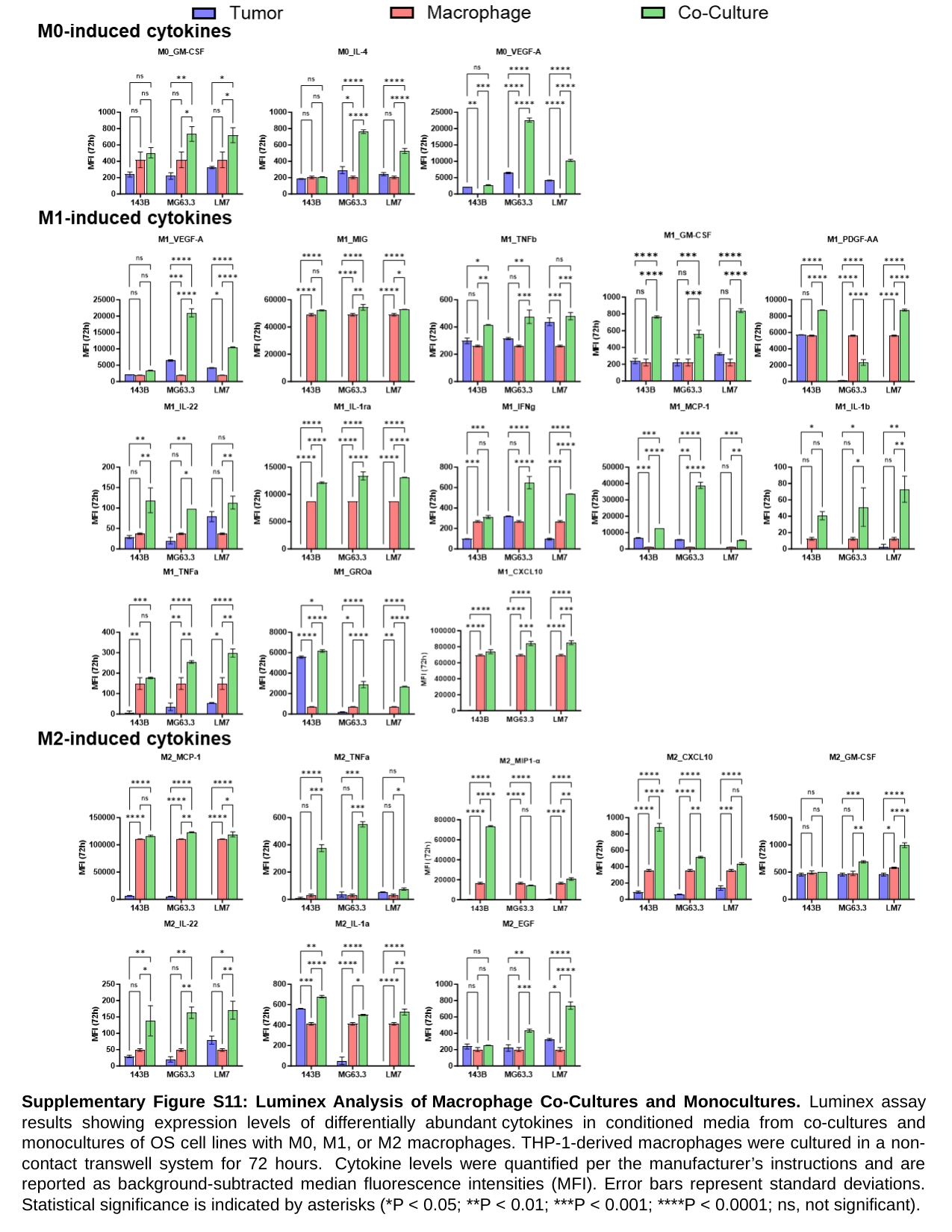

Supplementary Figure S11: Luminex Analysis of Macrophage Co-Cultures and Monocultures. Luminex assay results showing expression levels of differentially abundant cytokines in conditioned media from co-cultures and monocultures of OS cell lines with M0, M1, or M2 macrophages. THP-1-derived macrophages were cultured in a non-contact transwell system for 72 hours.  Cytokine levels were quantified per the manufacturer’s instructions and are reported as background-subtracted median fluorescence intensities (MFI). Error bars represent standard deviations. Statistical significance is indicated by asterisks (*P < 0.05; **P < 0.01; ***P < 0.001; ****P < 0.0001; ns, not significant).

## Slide 12
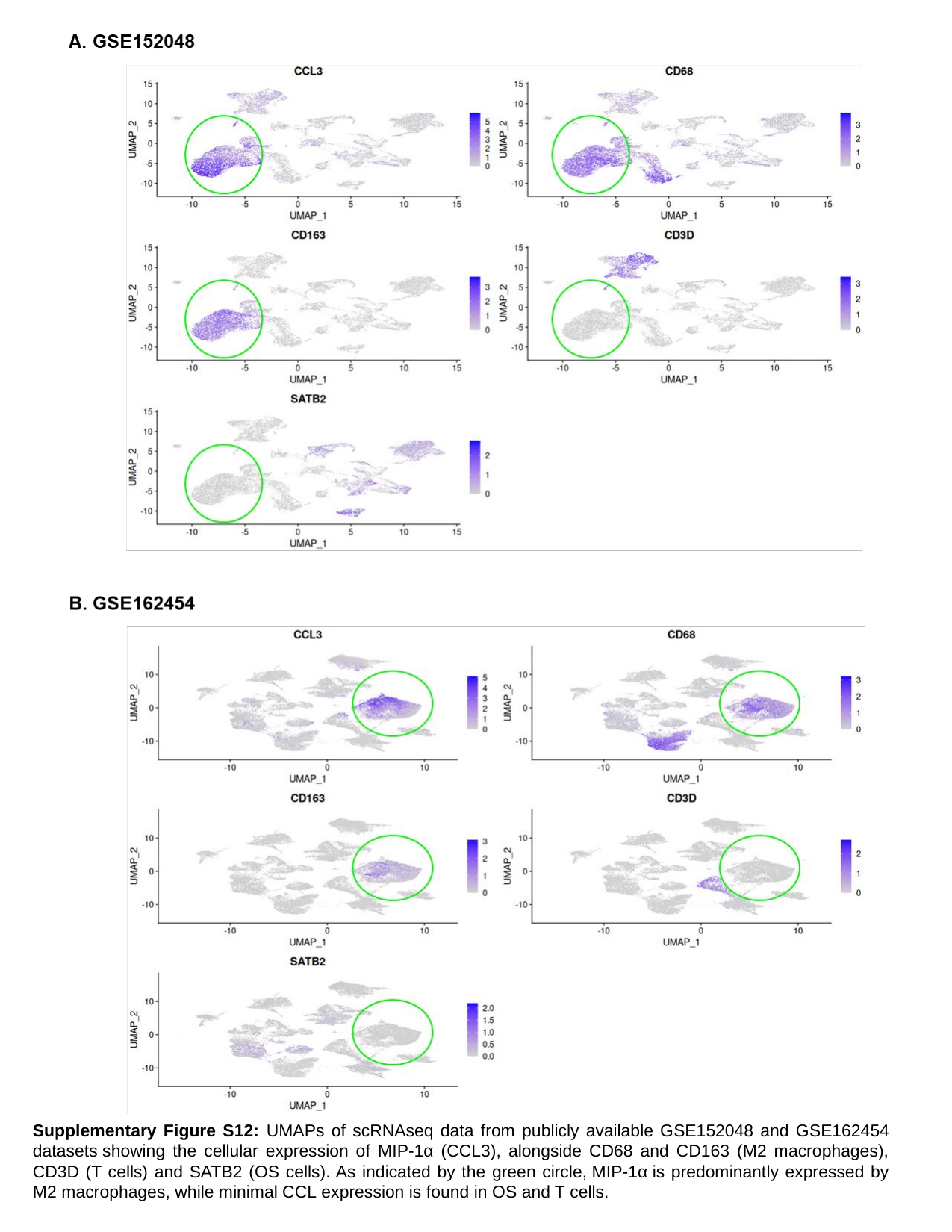

Supplementary Figure S12: UMAPs of scRNAseq data from publicly available GSE152048 and GSE162454 datasets showing the cellular expression of MIP-1α (CCL3), alongside CD68 and CD163 (M2 macrophages), CD3D (T cells) and SATB2 (OS cells). As indicated by the green circle, MIP-1α is predominantly expressed by M2 macrophages, while minimal CCL expression is found in OS and T cells.

## Slide 13
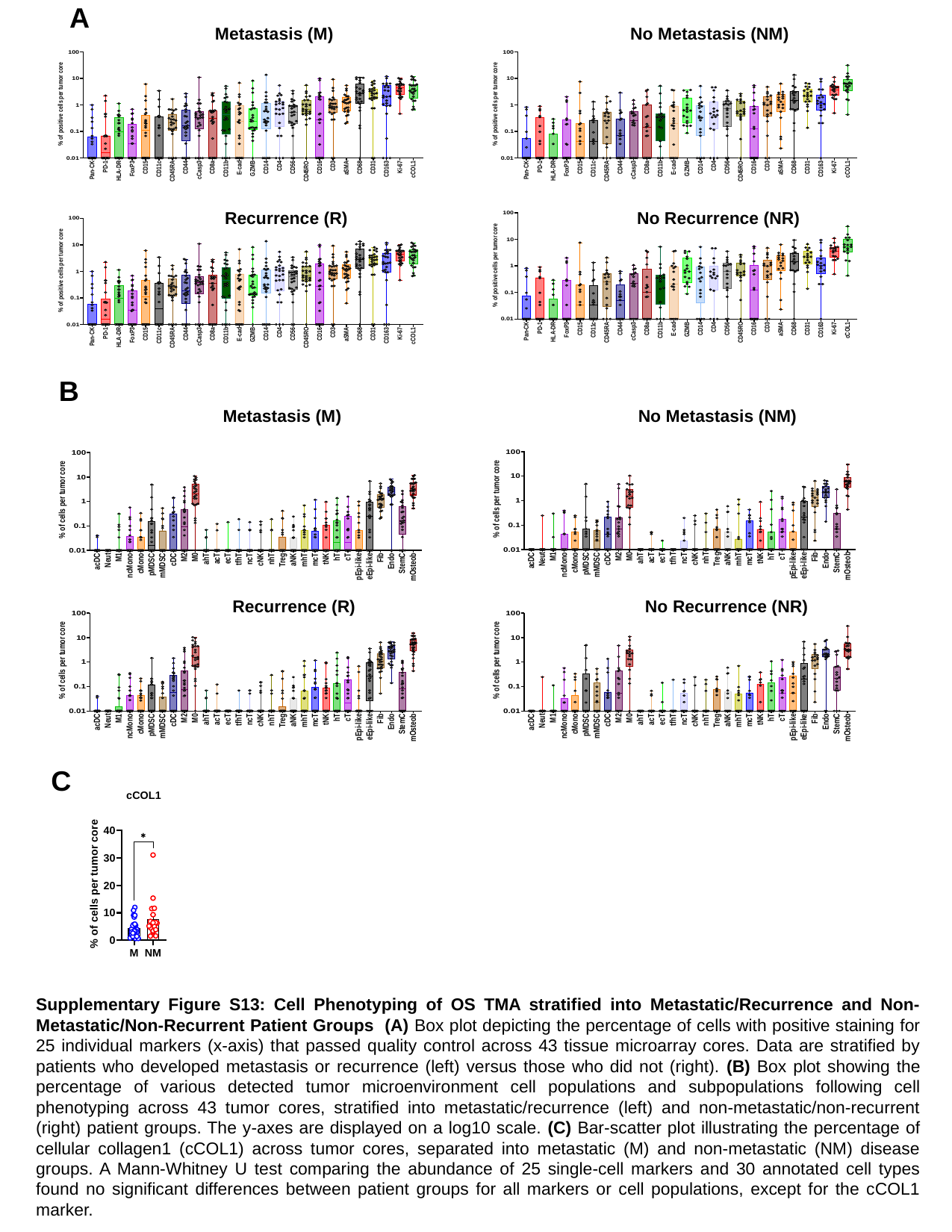

A
No Metastasis (NM)
Metastasis (M)
Recurrence (R)
No Recurrence (NR)
B
No Metastasis (NM)
Metastasis (M)
Recurrence (R)
No Recurrence (NR)
C
Supplementary Figure S13: Cell Phenotyping of OS TMA stratified into Metastatic/Recurrence and Non-Metastatic/Non-Recurrent Patient Groups (A) Box plot depicting the percentage of cells with positive staining for 25 individual markers (x-axis) that passed quality control across 43 tissue microarray cores. Data are stratified by patients who developed metastasis or recurrence (left) versus those who did not (right). (B) Box plot showing the percentage of various detected tumor microenvironment cell populations and subpopulations following cell phenotyping across 43 tumor cores, stratified into metastatic/recurrence (left) and non-metastatic/non-recurrent (right) patient groups. The y-axes are displayed on a log10 scale. (C) Bar-scatter plot illustrating the percentage of cellular collagen1 (cCOL1) across tumor cores, separated into metastatic (M) and non-metastatic (NM) disease groups. A Mann-Whitney U test comparing the abundance of 25 single-cell markers and 30 annotated cell types found no significant differences between patient groups for all markers or cell populations, except for the cCOL1 marker.

## Slide 14
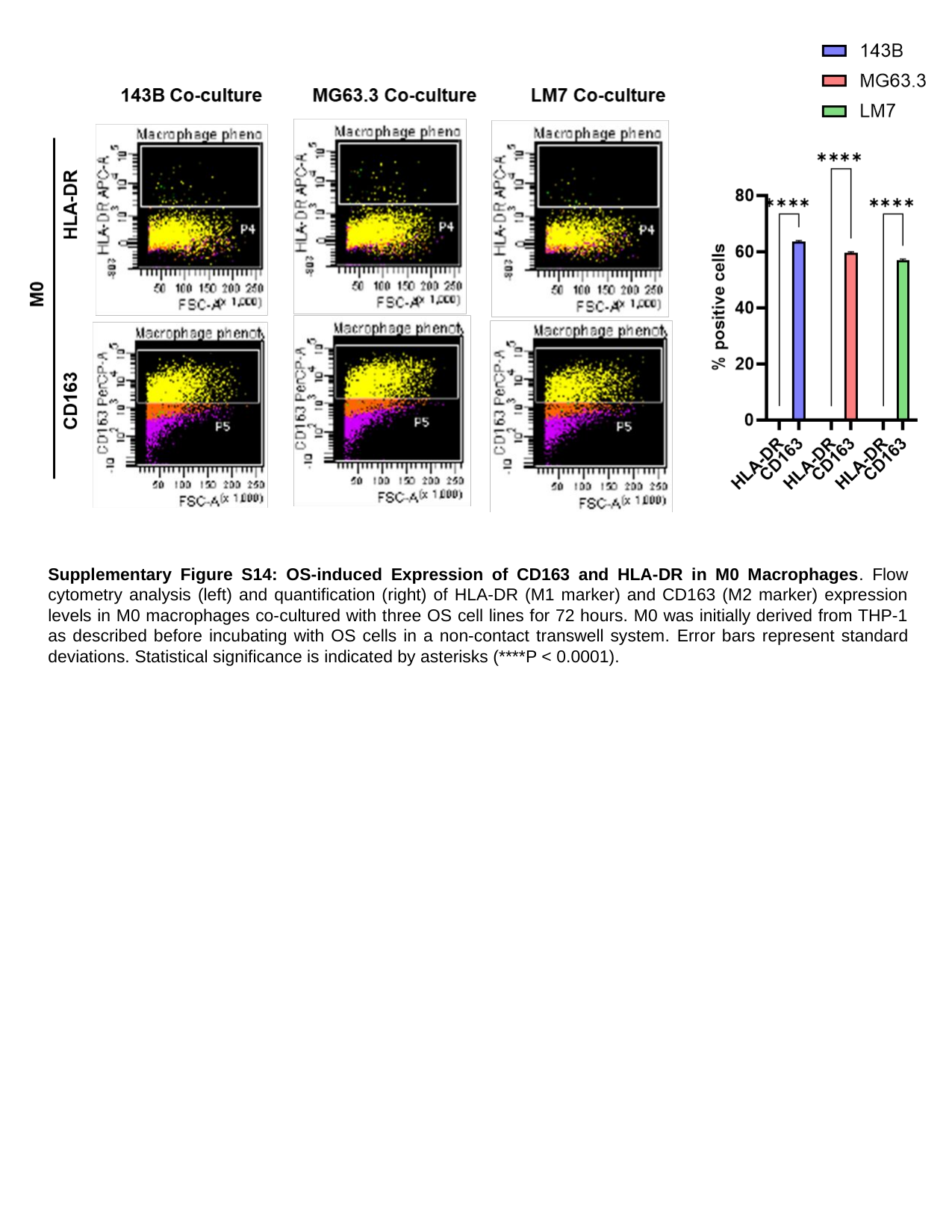

Supplementary Figure S14: OS-induced Expression of CD163 and HLA-DR in M0 Macrophages. Flow cytometry analysis (left) and quantification (right) of HLA-DR (M1 marker) and CD163 (M2 marker) expression levels in M0 macrophages co-cultured with three OS cell lines for 72 hours. M0 was initially derived from THP-1 as described before incubating with OS cells in a non-contact transwell system. Error bars represent standard deviations. Statistical significance is indicated by asterisks (****P < 0.0001).

## Slide 15
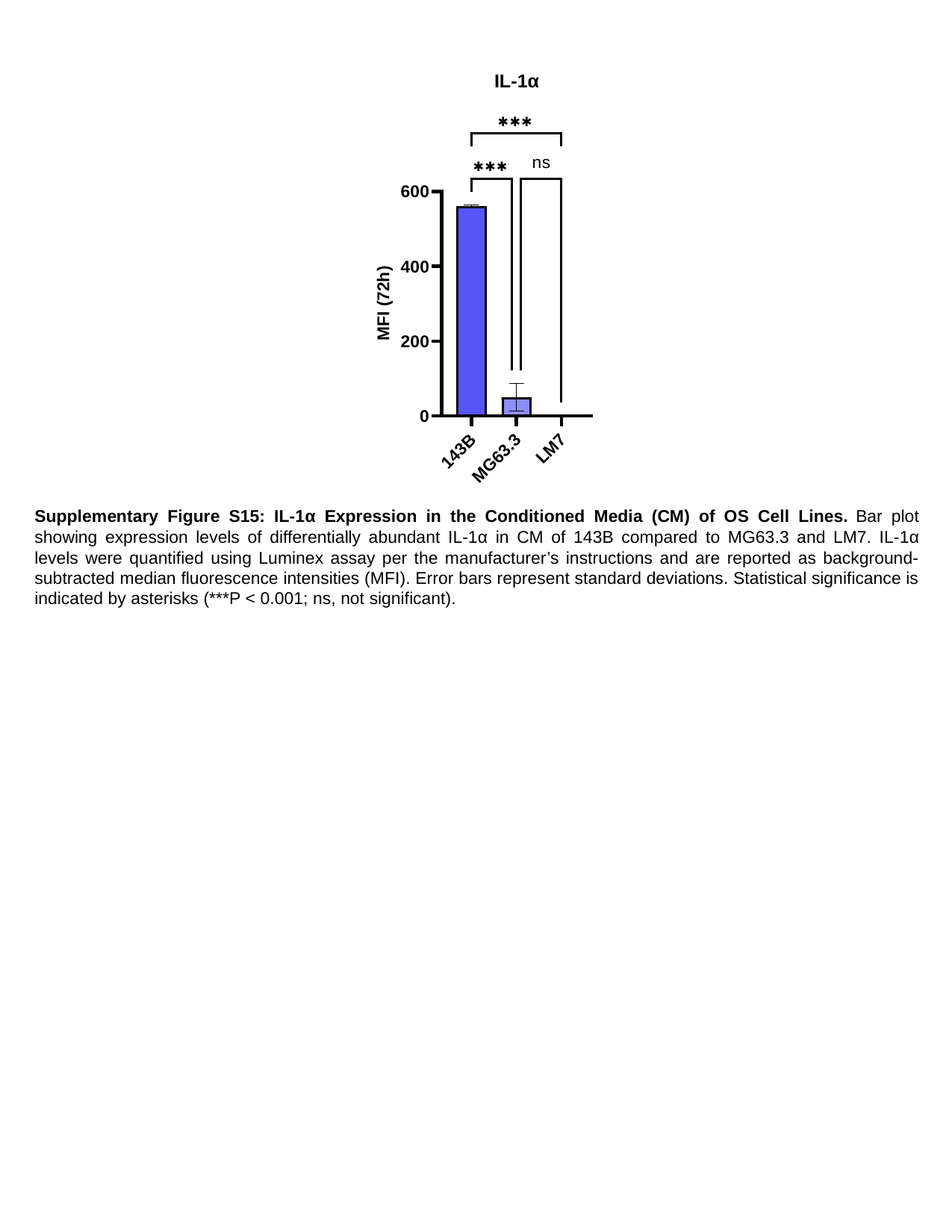

Supplementary Figure S15: IL-1α Expression in the Conditioned Media (CM) of OS Cell Lines. Bar plot showing expression levels of differentially abundant IL-1α in CM of 143B compared to MG63.3 and LM7. IL-1α levels were quantified using Luminex assay per the manufacturer’s instructions and are reported as background-subtracted median fluorescence intensities (MFI). Error bars represent standard deviations. Statistical significance is indicated by asterisks (***P < 0.001; ns, not significant).
